# Associations of height-for-age and BMI-for-age *Z*-scores with pediatric urolithiasis: a multicenter study

**DOI:** 10.3389/fnut.2026.1880262

**Published:** 2026-09-28

**Authors:** Daniyaer Nuerdebieke, Bide Liu, Xun Li, Shuheng Wang, Qiang Dong, Jiasuer Batuer, Honglin Yang, Kaisong Hu, Jiuzhi Li

**Affiliations:** 1 Urology Center, People’s Hospital of Xinjiang Uygur Autonomous Region, Urumqi, China; 2 Urology Center, The First Hospital of Kashgar Prefecture, Kashgar, China; 3 Urology Center, Yili Friendship Hospital, Yining, China

**Keywords:** BMI-for-age *Z*-score, growth impairment, height-for-age *Z*-score, nutritional status, pediatric urolithiasis

## Abstract

**Background:**

Pediatric urolithiasis is an increasing public health concern in high-prevalence regions, but the relationship between child growth status and the odds of stone disease remains unclear.

**Objective:**

This study aimed to evaluate the associations of height-for-age *Z*-score (HAZ) and BMI-for-age *Z*-score (BAZ) with pediatric urolithiasis in a large multicenter population from Xinjiang, China.

**Methods:**

This hospital-based retrospective multicenter case–control study included 14,027 children aged <15 years, comprising 2,180 children with imaging-confirmed renal and/or ureteral stones and 11,847 controls without urolithiasis. HAZ and BAZ were calculated according to World Health Organization growth standards and references. Multivariable logistic regression models were used to assess associations between HAZ, BAZ, and urolithiasis after adjustment for sex, ethnicity, age, and study center. Restricted cubic spline models were used to examine dose–response and nonlinear relationships, and subgroup analyses were performed by study center, sex, ethnicity, age group, and nutritional status.

**Results:**

Children with urolithiasis had lower HAZ and BAZ than controls (HAZ: −0.79 (−1.74, 0.24) compared with 0.57 (−0.29, 1.41); BAZ: −0.25 (−1.19, 0.77) compared with 0.36 (−0.61, 1.43); both *p* < 0.001). After full adjustment, each 1-unit increase in HAZ was associated with 36% lower odds of urolithiasis (odds ratio: 0.64; 95% CI: 0.61, 0.66; *p* < 0.001), and each 1-unit increase in BAZ was associated with 21% lower odds (odds ratio: 0.79; 95% CI: 0.76, 0.81; *p* < 0.001). HAZ showed a nonlinear inverse association with the estimated odds of urolithiasis, whereas BAZ showed an approximately linear inverse association. Subgroup analyses showed that the inverse associations were generally consistent across sexes and ethnicities. For HAZ, significant interactions were observed by study center, age group, and BAZ-defined nutritional status, whereas BAZ showed more stable associations across most subgroups.

**Conclusion:**

Lower HAZ and BAZ were associated with higher odds of pediatric urolithiasis, with HAZ showing a stronger and nonlinear association.

## Introduction

Pediatric kidney stones represent an increasingly important global health problem, with incidence rates rising over the past several decades (1, 2). Compared with adults, children may experience acute pain, urinary tract infection, recurrent disease, and longer-term kidney or psychosocial consequences (3–6). Epidemiological studies have shown substantial geographic and ethnic variation, including high burdens in several endemic regions. Because childhood is a critical period of growth and development, nutritional status, metabolic characteristics, and growth patterns may be associated with stone occurrence (7, 8). Careful evaluation of these associations may help define priorities for prospective research.

Height-for-age *Z*-score (HAZ) and BMI-for-age *Z*-score (BAZ) are standardized indicators for assessing child growth and nutritional status (9). HAZ reflects linear growth and long-term nutritional conditions, with low HAZ values indicating stunting or chronic malnutrition. BAZ evaluates the proportional relationship between weight and height, identifying nutritional imbalances such as wasting, overweight, or obesity (10, 11). These two indicators have been widely recommended by the World Health Organization for child health monitoring and have been demonstrated in multiple studies to be closely associated with metabolic syndrome and cardiovascular disease risk (12–14). However, the relationship between growth indicators and pediatric kidney stones remains inadequately elucidated.

Evidence on body size and pediatric urolithiasis is mixed. Some studies of pediatric stone formers have linked overweight or obesity to lower urine volume, hyperuricosuria, or other lithogenic profiles, whereas primary-care case–control data have not consistently shown higher stone odds with greater BMI (15–19). This heterogeneity may reflect differences in age, nutritional context, stone composition, selection mechanisms, and healthcare setting. Xinjiang is a high-burden, multi-ethnic region with distinctive environmental and dietary contexts (20), making it useful for examining how standardized growth indicators relate to pediatric urolithiasis while recognizing that the findings may not generalize to other settings.

Using multicenter child-health data from Xinjiang, we evaluated the associations of HAZ and BAZ with pediatric urolithiasis. We used multivariable logistic regression, restricted cubic splines, subgroup analyses, and design-based sensitivity analyses to characterize the magnitude, shape, robustness, and heterogeneity of these associations. The study was intended to generate epidemiologic evidence rather than to establish causality or a clinical screening threshold.

## Methods

### Study population

This hospital-based retrospective multicenter case–control study was conducted to investigate the associations of HAZ and BAZ with pediatric urolithiasis. Cases and controls were enrolled contemporaneously from the same participating institutions during the same study period (2011–2025). The three participating hospitals were grouped into two analytic centers for statistical analysis: Center A (the People’s Hospital of Xinjiang Uygur Autonomous Region, Urumqi) and Center B (the First People’s Hospital of Kashgar Prefecture and Yili Friendship Hospital, combined), reflecting *a priori* geographic and demographic clustering. Children diagnosed with urolithiasis between January 2011 and November 2025 were included as cases, whereas children who underwent routine health examinations during the same period and had no history or symptoms of urolithiasis were included as controls.

Eligible cases were children aged <15 years with renal and/or ureteral stones confirmed by ultrasonography, non-contrast computed tomography, or another clinically accepted imaging modality. The database-construction rule required demographic data, documented anthropometric measurements, and a prespecified core laboratory panel used for descriptive characterization; records lacking any of these required fields were excluded during screening (Figure 1). Cases were excluded for definite urinary tract anatomical abnormalities, severe systemic disease or malignant tumor, major prior urinary tract surgery, or missing required fields.

**Figure 1:**
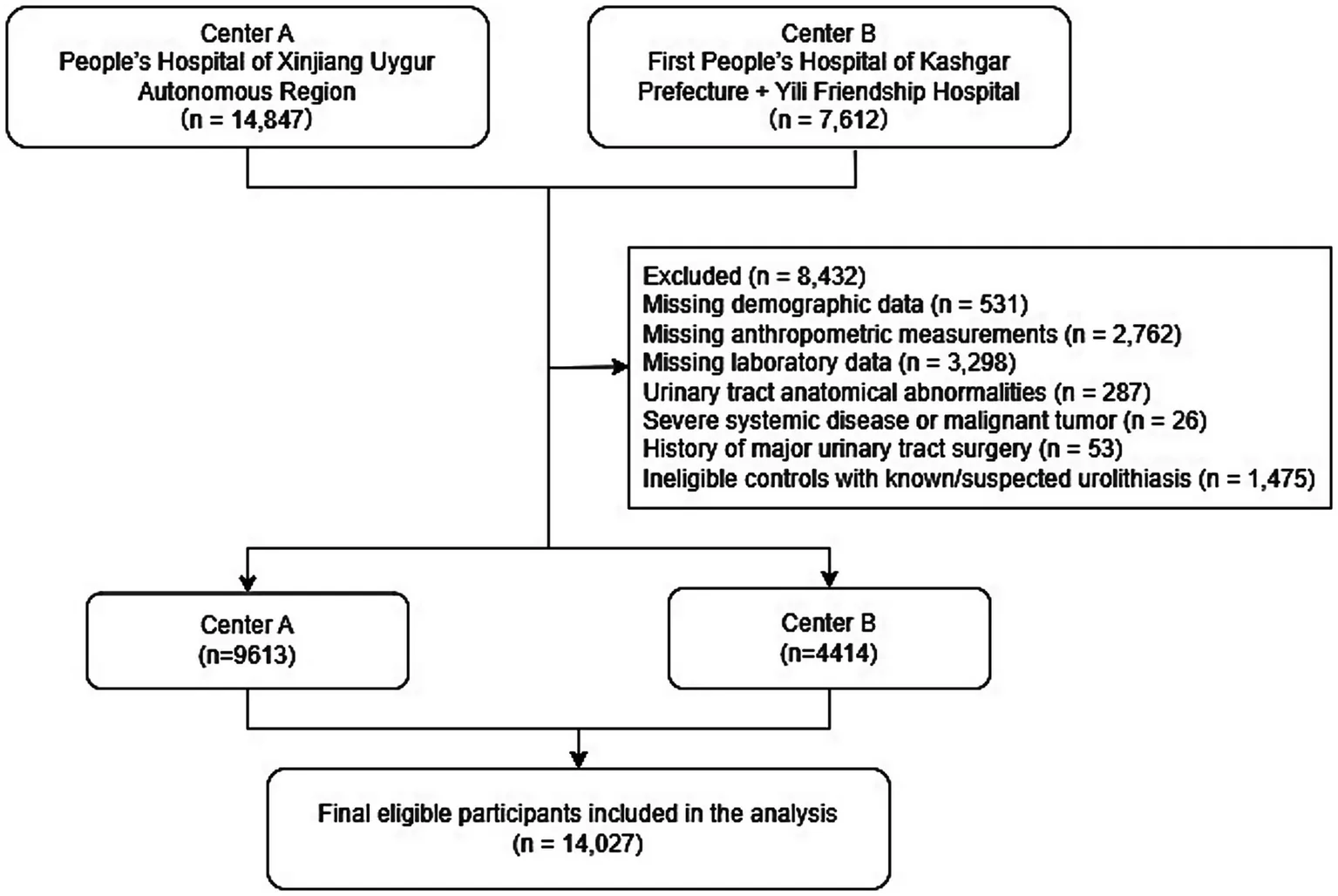
Flowchart of participant inclusion.

Controls were children aged <15 years who attended health examinations at the participating hospitals during the same period and had no documented history or symptoms suggestive of urolithiasis. The same completeness rule for demographic, anthropometric, and prespecified laboratory fields was applied. Controls with known or suspected urolithiasis, chronic kidney disease, severe systemic disease, malignant tumor, or missing required fields were excluded, as shown in Figure 1.

Participant flow: A total of 22,459 records were screened (Center A, *n* = 14,847; Center B, *n* = 7,612). We excluded 8,432 records because of missing demographic data (*n* = 531), missing anthropometric measurements (*n* = 2,762), missing laboratory data (*n* = 3,298; all were health-examination children who had completed neither urinalysis nor blood testing at the qualifying encounter), urinary tract anatomical abnormalities (*n* = 287), severe systemic disease or malignant tumor (*n* = 26), major urinary tract surgery (*n* = 53), or known/suspected urolithiasis among potential controls (*n* = 1,475). The final analytic cohort comprised 14,027 children (2,180 cases and 11,847 controls; Center A, *n* = 9,613; Center B, *n* = 4,414; Figure 1; Supplementary Table S12).

### Primary exposure variables

HAZ and BAZ were the primary exposure variables in this study. HAZ and BAZ were calculated using WHO Anthro software for children aged 0–5 years according to the WHO Child Growth Standards, and WHO AnthroPlus software for children and adolescents aged 5–19 years according to the WHO 2007 growth reference. HAZ reflects linear growth and long-term nutritional status of children. Stunting was defined according to WHO standards: severe stunting (HAZ < -3), moderate stunting (−3 ≤ HAZ < -2), and non-stunting (HAZ ≥ -2). BAZ evaluates the proportional relationship between weight and height. According to WHO standards, children were classified as thinness (BAZ < −2), normal (−2 ≤ BAZ ≤ 1), overweight (1 < BAZ ≤ 2), and obesity (BAZ > 2). In subgroup analyses, overweight and obesity were combined as overweight/obesity when appropriate (9).

### Covariates

Collected covariates included: (1) demographic characteristics: sex (male/female), ethnicity (Uyghur/Han/Kazakh/Other), age (months), and study center (A/B); (2) blood routine parameters: red blood cell count (RBC), mean corpuscular volume (MCV), mean corpuscular hemoglobin concentration (MCHC), mean corpuscular hemoglobin (MCH), red cell distribution width-coefficient of variation (RDW-CV), hematocrit (HCT), hemoglobin (Hb), platelet count (PLT), mean platelet volume (MPV), platelet distribution width (PDW), etc.; (3) urinalysis parameters: urinary protein, urinary bilirubin, urinary glucose, urinary ketone bodies, specific gravity (SG), urinary pH, etc.; (4) blood biochemistry parameters: alanine aminotransferase (ALT), aspartate aminotransferase (AST), gamma-glutamyl transferase (GGT), direct bilirubin (DBIL), total bilirubin (TBIL), albumin (ALB), indirect bilirubin (IBIL), total protein (TP), globulin (GLO), creatinine (CREA), blood urea nitrogen (BUN), uric acid (UA), potassium (K), chloride (Cl), sodium (Na), magnesium (Mg), calcium (Ca), cholinesterase (CHE), prothrombin time (PT), activated partial thromboplastin time (APTT), fibrinogen (FBG), D-dimer, etc. Hematological, urinary, and biochemical measurements were performed in the clinical laboratories of the participating hospitals as part of routine clinical care, using automated analyzers under each hospital’s standard operating procedures and internal quality-control programs. Because of the retrospective design and the 14-year study period, the specific analyzer platforms, assay reagents, and calibration records could not be verified across centers and over time; this is acknowledged as a limitation.

Anthropometric measurements: Height and weight values were extracted from routine electronic medical records. For cases, the earliest measurements documented during the admission encounter were used; for controls, measurements from the health-examination visit were used. The specific equipment, staff-training records, and uniformity of measurement protocols could not be verified across all centers and years. HAZ and BAZ were calculated using age- and sex-specific WHO references; this standardization facilitates comparison by age and sex but does not correct measurement error or systematic differences in measurement protocols. Biologically implausible *Z*-scores were handled according to WHO flagging criteria (|HAZ| > 6; |BAZ| > 5). In the primary analysis dataset, 5 records (0.04%) had HAZ or BAZ values exceeding these thresholds; a sensitivity analysis excluding these records yielded virtually identical estimates (joint model: HAZ OR 0.591, 95% CI: 0.570–0.613; BAZ OR 0.703, 95% CI: 0.678–0.729).

### Statistical analysis

All statistical analyses were performed using R software (version 4.5.3) with the rms, car, MatchIt, WeightIt, cobalt, survey, pROC, ResourceSelection, ggplot2, and dplyr packages. Continuous variables were presented as median (interquartile range), and categorical variables as number (percentage). Between-group comparisons were performed using the Mann–Whitney U test for continuous variables and the chi-square test for categorical variables. Standardized mean differences (SMDs) were calculated to assess between-group differences, with SMD > 0.1 considered clinically meaningful.

Multivariable logistic regression analyses were used to evaluate the associations of HAZ and BAZ with pediatric urolithiasis. Three models were constructed: Model 1 was unadjusted; Model 2 adjusted for sex, ethnicity, and age in months; and Model 3 further adjusted for study center. HAZ and BAZ were first modeled separately as continuous variables. To assess their independent associations, HAZ and BAZ were then entered simultaneously into a joint multivariable logistic regression model. Multicollinearity was evaluated using Pearson and Spearman correlation coefficients and variance inflation factors. No *a priori* sample size calculation was performed, as this retrospective study included all eligible records during the study period. Post-hoc calculations indicated that, with 2,180 cases and 11,847 controls, the study had >99.9% power to detect the observed HAZ association (OR 0.64 per 1 unit) at a two-sided α of 0.05, and an odds ratio as small as 0.958 per unit would have been detectable with 80% power.

Blood and urine biochemical parameters were excluded from the primary etiologic models because they may lie on the causal pathway between nutritional status and stone formation or may act as colliders, and adjusting for them could introduce overadjustment bias. These parameters were used for descriptive characterization only; those with substantial missingness were imputed and their between-group comparisons should be interpreted with caution.

To address potential imbalances in study center and ethnicity between cases and controls, additional center-specific, ethnicity-specific, and center-by-ethnicity stratified analyses were performed. Propensity score matching and propensity score weighting were also conducted as sensitivity analyses. The propensity score model included study center, ethnicity, sex, and age, whereas HAZ and BAZ were not included because they were the primary exposure variables. Covariate balance was assessed using SMDs, with values below 0.1 considered acceptable. Cases were matched 1:1 to controls using nearest-neighbor matching without replacement, with exact matching on study center and ethnicity and a caliper of 0.2 standard deviations of the logit of the propensity score. In the matched sample, a joint logistic regression model was fitted with pair-clustered design-based standard errors and adjustment for the matching covariates. As complementary approaches, stabilized inverse probability of treatment weighting (average treatment effect) and overlap weighting (average treatment effect in the overlap population) were applied, followed by weighted joint logistic regression with robust sandwich standard errors. In this case–control application, the “treatment” in the weighting estimands refers to case–control group membership and is used solely as a design device to balance measured covariates between the two groups; no treatment effect is estimated. The average treatment effect (ATE) weights target balance in the full analytic sample, whereas overlap (ATO) weights target the subpopulation with clinical equipoise, that is, overlapping covariate distributions.

Restricted cubic spline models were used to evaluate dose–response and nonlinear associations. Four knots were placed at the 5th, 35th, 65th, and 95th percentiles, and the exact knot locations were reported in the Supplementary materials. The significance of nonlinear associations was assessed using likelihood ratio tests. Subgroup analyses were conducted by study center, sex, ethnicity, age group, and nutritional status. Interaction terms were included in multivariable logistic regression models to test for subgroup interactions. Subgroup interaction *p*-values were additionally corrected for multiple testing using the false discovery rate method and Bonferroni correction. All statistical tests were two-sided, with *p* < 0.05 considered statistically significant. Model performance was evaluated using the C-statistic (area under the receiver operating characteristic curve) with DeLong 95% confidence intervals, calibration intercept and slope, the Brier score, the Hosmer–Lemeshow test, and the Akaike and Bayesian information criteria (Supplementary Table S13).

### Missing data

Eligibility screening required demographic data, documented anthropometric measurements, and a prespecified core laboratory panel comprising urinalysis and routine blood testing. The 3,298 records excluded for missing laboratory data were all from the health-examination (potential control) population: these children had completed neither urinalysis nor blood testing at the qualifying health-examination encounter. No cases were excluded for this reason, because laboratory evaluation is performed routinely during hospitalization. Within the retained analytic sample (*N* = 14,027), sporadic item-level missing data remained: 0% for the outcome, sex, ethnicity, and age; 1.95% for HAZ and 2.57% for BAZ; and 0.2–32.9% across secondary laboratory variables (Supplementary Table S12). These missing values were handled by multiple imputation by chained equations (MICE) with 20 imputed datasets, using predictive mean matching for continuous variables; imputation models included the outcome and all model covariates, and pooled estimates were combined using Rubin’s rules. Missingness of several laboratory panels was more frequent among controls than among cases, reflecting more extensive laboratory workups during hospitalization; case status was therefore included in all imputation models. Laboratory variables with substantial missingness (e.g., urinary protein 32.9%, magnesium 27.2%, cholinesterase 26.7%, calcium 21.0%) were used for descriptive characterization only and were not included in any inferential model. Estimates from the primary analysis dataset and from MICE-pooled sensitivity analyses were materially consistent (joint model, MICE-pooled: HAZ OR 0.607, 95% CI: 0.586–0.629; BAZ OR 0.729, 95% CI: 0.702–0.757).

## Results

### Baseline characteristics of study participants

This study enrolled 14,027 children, including 2,180 (15.54%) in the kidney stone group and 11,847 (84.46%) in the non-stone group. Table 1 presents comparisons of baseline characteristics, blood routine parameters, urinalysis, and blood biochemistry indicators between the two groups. Regarding baseline characteristics, the proportion of females was higher in the stone group compared to the non-stone group (39.04% vs. 34.52%, *p* < 0.001). Ethnic distribution showed significant differences (*p* < 0.001), with Uyghur children accounting for 92.61% of the stone group but only 51.52% of the non-stone group. The median age in the stone group was 74 months, slightly lower than 80 months in the non-stone group (*p* < 0.001).

**Table 1:** Comparison of baseline characteristics and clinical parameters between study groups.

Variable	Total (*n* = 14,027)	Non-stone (*n* = 11,847)	Stone (*n* = 2,180)	Statistic	*p*	SMD
Gender, *n* (%)				*χ*^2^ = 16.436	<0.001	
Male	9,086 (64.78)	7,757 (65.48)	1,329 (60.96)			−0.093
Female	4,941 (35.22)	4,090 (34.52)	851 (39.04)			0.093
Ethnicity, *n* (%)				*χ*^2^ = 1293.610	<0.001	
Uyghur	8,122 (57.9)	6,103 (51.52)	2019 (92.61)			1.571
Han	4,257 (30.35)	4,185 (35.33)	72 (3.30)			−1.792
Kazakh	912 (6.5)	846 (7.14)	66 (3.03)			−0.240
Other	736 (5.25)	713 (6.02)	23 (1.06)			−0.486
Age (month)	80.00 (53.00, 114.00)	80.00 (55.00, 113.00)	74.00 (30.00, 119.00)	*Z* = −7.086	<0.001	−0.115
HAZ	0.40 (−0.54, 1.30)	0.57 (−0.29, 1.41)	−0.79 (−1.74, 0.24)	*Z* = −35.801	<0.001	−0.760
BAZ	0.28 (−0.68, 1.34)	0.36 (−0.61, 1.43)	−0.25 (−1.19, 0.77)	*Z* = −17.114	<0.001	−0.426
RBC	4.74 (4.49, 5.00)	4.77 (4.53, 5.02)	4.57 (4.29, 4.85)	*Z* = −20.665	<0.001	0.002
MCV	82.40 (79.40, 85.30)	82.70 (79.90, 85.50)	80.50 (76.40, 83.90)	*Z* = −19.822	<0.001	−0.419
MCHC	329.00 (320.00, 338.00)	329.00 (321.00, 338.00)	328.00 (317.00, 338.00)	*Z* = −5.843	<0.001	−0.139
MCH	27.20 (26.10, 28.20)	27.30 (26.30, 28.30)	26.50 (24.70, 27.80)	*Z* = −19.054	<0.001	−0.393
RDW-CV	13.20 (12.60, 13.80)	13.10 (12.60, 13.60)	13.60 (12.90, 14.90)	*Z* = −23.664	<0.001	0.482
HCT	0.39 (0.37, 0.42)	0.40 (0.38, 0.42)	0.37 (0.34, 0.39)	*Z* = −34.513	<0.001	−0.817
Hb	128.00 (121.00, 135.00)	130.00 (123.00, 136.00)	120.04 (111.00, 128.00)	*Z* = −32.120	<0.001	−0.356
PLT	331.00 (282.00, 388.00)	329.00 (281.00, 381.00)	351.00 (288.00, 433.00)	*Z* = −10.874	<0.001	0.277
MPV	10.10 (9.20, 16.90)	10.00 (9.20, 16.50)	10.50 (9.10, 18.20)	*Z* = −2.949	0.003	0.099
PDW	11.70 (10.10, 15.30)	11.50 (10.10, 15.30)	12.70 (10.40, 15.40)	*Z* = −7.982	<0.001	0.231
PLB	0.32 (0.27, 0.37)	0.31 (0.27, 0.36)	0.33 (0.28, 0.39)	*Z* = −8.761	<0.001	0.224
SD	39.40 (37.70, 41.40)	39.40 (37.70, 41.20)	39.70 (37.70, 42.30)	*Z* = −6.889	<0.001	0.202
U Tp, *n* (%)				*χ*^2^ = 4649.855	<0.001	
Positive	1,259 (8.98)	227 (1.92)	1,032 (47.34)			0.910
Negative	12,768 (91.02)	11,620 (98.08)	1,148 (52.66)			−0.910
URO, *n* (%)				*χ*^2^ = 21.700	<0.001	
Positive	408 (2.91)	311 (2.63)	97 (4.45)			0.088
Negative	13,619 (97.09)	11,536 (97.37)	2083 (95.55)			−0.088
BIL, *n* (%)				*χ*^2^ = 58.526	<0.001	
Positive	200 (1.43)	130 (1.10)	70 (3.21)			0.120
Negative	13,827 (98.57)	11,717 (98.90)	2,110 (96.79)			−0.120
Glu, *n* (%)				*χ*^2^ = 212.926	<0.001	
Positive	70 (0.5)	15 (0.13)	55 (2.52)			0.153
Negative	13,957 (99.5)	11,832 (99.87)	2,125 (97.48)			−0.153
KET, *n* (%)				*χ*^2^ = 431.199	<0.001	
Positive	444 (3.17)	219 (1.85)	225 (10.32)			0.278
Negative	13,583 (96.83)	11,628 (98.15)	1955 (89.68)			−0.278
Sg	1.02 (1.02, 1.03)	1.02 (1.02, 1.03)	1.02 (1.01, 1.02)	*Z* = −30.634	<0.001	−1.459
Ph	5.88 (5.50, 6.00)	5.87 (5.50, 6.00)	6.00 (5.50, 6.03)	*Z* = −4.336	<0.001	0.092
ALT	14.00 (11.00, 19.00)	14.00 (11.00, 19.00)	15.00 (11.30, 20.43)	*Z* = −4.657	<0.001	0.083
AST	25.00 (21.60, 30.00)	25.00 (21.70, 30.00)	26.00 (21.05, 32.00)	*Z* = −4.497	<0.001	0.021
GGT	12.00 (10.00, 14.00)	12.00 (10.24, 14.00)	12.00 (10.00, 14.00)	*Z* = −4.276	<0.001	0.076
Dbil	2.40 (1.80, 3.20)	2.44 (1.80, 3.28)	2.10 (1.59, 2.87)	*Z* = −12.949	<0.001	−0.049
TBIL	6.80 (5.20, 9.30)	6.99 (5.30, 9.50)	5.95 (4.58, 7.93)	*Z* = −15.041	<0.001	−0.106
ALB	43.00 (40.89, 45.10)	43.10 (41.00, 45.19)	42.17 (39.80, 44.61)	*Z* = −11.592	<0.001	−0.215
IBIL	4.21 (2.89, 6.00)	4.37 (3.00, 6.14)	3.60 (2.30, 5.06)	*Z* = −14.952	<0.001	−0.138
Tp	68.90 (65.40, 72.60)	69.30 (65.90, 72.90)	66.24 (62.88, 70.37)	*Z* = −21.374	<0.001	−0.146
Glo	25.30 (21.66, 28.40)	25.55 (21.98, 28.50)	24.00 (20.70, 27.40)	*Z* = −8.172	<0.001	0.014
CREA	35.00 (29.00, 41.50)	35.00 (29.40, 41.02)	34.00 (26.70, 44.10)	*Z* = −1.614	0.107	0.091
BUN	4.40 (3.62, 5.24)	4.34 (3.60, 5.17)	4.69 (3.77, 5.77)	*Z* = −10.633	<0.001	0.043
UA	240.26 (200.96, 288.66)	243.00 (204.00, 291.00)	222.00 (185.35, 269.59)	*Z* = −12.304	<0.001	−0.029
K^+^	4.32 (4.10, 4.56)	4.33 (4.12, 4.57)	4.22 (3.97, 4.51)	*Z* = −11.935	<0.001	0.026
Cl^−^	105.00 (103.20, 107.00)	105.00 (103.58, 107.00)	104.80 (102.10, 106.90)	*Z* = −8.626	<0.001	−0.213
Na^+^	139.40 (138.00, 141.00)	139.70 (138.00, 141.00)	138.80 (136.97, 140.02)	*Z* = −16.874	<0.001	−0.272
Mg^2+^	0.87 (0.82, 0.92)	0.87 (0.82, 0.91)	0.86 (0.80, 0.93)	*Z* = −3.055	0.002	0.022
Ca^2+^	2.40 (2.33, 2.47)	2.41 (2.34, 2.47)	2.37 (2.28, 2.47)	*Z* = −12.938	<0.001	−0.250
Che	9.00 (8.00, 10.00)	9.17 (8.29, 10.14)	7.86 (6.70, 8.86)	*Z* = −34.711	<0.001	−0.757
PT Inr	1.03 (0.98, 1.08)	1.03 (0.97, 1.08)	1.04 (0.98, 1.10)	*Z* = −7.461	<0.001	0.129
PT	11.90 (11.30, 12.80)	11.90 (11.30, 12.60)	12.50 (11.60, 13.50)	*Z* = −19.467	<0.001	0.308
PTR	1.04 (0.98, 1.11)	1.03 (0.98, 1.10)	1.07 (1.00, 1.17)	*Z* = −12.803	<0.001	0.247
PTA	94.00 (86.00, 102.80)	95.00 (86.60, 103.00)	91.00 (83.10, 99.00)	*Z* = −12.490	<0.001	−0.291
TT	17.40 (16.14, 18.59)	17.50 (16.10, 18.60)	17.20 (16.20, 18.30)	*Z* = −3.142	0.002	−0.038
APTT	31.00 (28.50, 35.50)	30.90 (28.50, 35.10)	32.21 (28.50, 38.20)	*Z* = −8.557	<0.001	0.218
FBG	2.47 (2.14, 2.89)	2.43 (2.12, 2.82)	2.76 (2.27, 3.39)	*Z* = −20.119	<0.001	0.448
D-Dimer	0.23 (0.15, 0.35)	0.22 (0.15, 0.33)	0.32 (0.20, 0.57)	*Z* = −22.348	<0.001	0.170

Continuous variables are presented as median (interquartile range), and categorical variables as number (percentage). Between-group comparisons were performed using Mann–Whitney U test (continuous variables) or chi-square test (categorical variables). SMD, standardized mean difference; HAZ, height-for-age *Z*-score; BAZ, BMI-for-age *Z*-score; RBC, red blood cell count; MCV, mean corpuscular volume; MCHC, mean corpuscular hemoglobin concentration; MCH, mean corpuscular hemoglobin; RDW-CV, red cell distribution width-coefficient of variation; HCT, hematocrit; Hb, hemoglobin; PLT, platelet count; MPV, mean platelet volume; PDW, platelet distribution width; U-TP, urinary protein; SG, specific gravity; ALT, alanine aminotransferase; AST, aspartate aminotransferase; GGT, gamma-glutamyl transferase; DBIL, direct bilirubin; TBIL, total bilirubin; ALB, albumin; IBIL, indirect bilirubin; TP, total protein; GLO, globulin; CREA, creatinine; BUN, blood urea nitrogen; UA, uric acid; CHE, cholinesterase; PT, prothrombin time; APTT, activated partial thromboplastin time; FBG, fibrinogen.

For growth indicators, both HAZ and BAZ were significantly lower in the stone group compared to the non-stone group. The median HAZ in the stone group was −0.79 (−1.74, 0.24) versus 0.57 (−0.29, 1.41) in the non-stone group (*p* < 0.001, SMD = −0.760); the median BAZ was −0.25 (−1.19, 0.77) in the stone group versus 0.36 (−0.61, 1.43) in the non-stone group (*p* < 0.001, SMD = −0.426).

Blood routine parameters revealed that red blood cell count (RBC), mean corpuscular volume (MCV), mean corpuscular hemoglobin (MCH), hematocrit (HCT), and hemoglobin (Hb) were all significantly lower in the stone group compared to the non-stone group (all *p* < 0.001), while red cell distribution width (RDW-CV) and platelet-related parameters (PLT, PDW) were significantly elevated (all *p* < 0.001).

Urinalysis demonstrated that the positive rate of urinary protein in the stone group was 47.34%, significantly higher than 1.92% in the non-stone group (*p* < 0.001). Additionally, positive rates of urinary bilirubin, glucose, and ketone bodies were all significantly higher in the stone group (all *p* < 0.001), while specific gravity was significantly lower (*p* < 0.001).

Regarding blood biochemistry, albumin (Alb), total protein (TP), cholinesterase (CHE) levels were all significantly lower in the stone group compared to the non-stone group (all *p* < 0.001), while blood urea nitrogen (BUN), prothrombin time (PT), fibrinogen (FBG), and D-dimer levels were significantly elevated (all *p* < 0.001).

A total of 14,027 children were included in this study, with 9,613 (68.53%) from Center A and 4,414 (31.47%) from Center B. The proportion of children with urolithiasis in this hospital-based case–control sample was 15.54% (*n* = 2,180), with comparable proportions between Center A (15.27%) and Center B (16.13%, *p* = 0.192). Males accounted for 64.78% of the study population, and Uyghur children represented the largest ethnic group (57.9%), followed by Han (30.35%), Kazakh (6.5%), and other ethnicities (5.25%). The median age was 80.00 months (IQR: 53.00–114.00), with children from Center B being significantly older than those from Center A (97.00 vs. 71.00 months, *p* < 0.001). The median HAZ and BAZ values were 0.40 (IQR: −0.54-1.30) and 0.28 (IQR: −0.68-1.34), respectively. Notable differences were observed between the two centers in several hematological, urinary, and biochemical parameters (all *p* < 0.05), reflecting potential regional and demographic heterogeneity, which justified the inclusion of study center as a key adjustment variable in subsequent analyses (Supplementary Table S1). Because marked ethnic imbalance was observed between the stone and non-stone groups, we performed additional stratified and propensity score-based sensitivity analyses to evaluate the robustness of the associations.

### Association between HAZ, BAZ and pediatric kidney stones

Table 2 presents the results of multivariable logistic regression analyses examining the associations between HAZ, BAZ and pediatric kidney stones. In the unadjusted model (Model 1), each 1-unit increase in BAZ was associated with 24% lower odds of kidney stones (OR = 0.76, 95%CI: 0.74–0.78, *p* < 0.001), with a significant dose–response relationship (*p* for trend <0.001). Each 1-unit increase in HAZ was associated with 43% lower odds of kidney stones (OR = 0.57, 95%CI: 0.55–0.59, *p* < 0.001), also demonstrating a significant dose–response relationship (*p* for trend <0.001).

**Table 2:** Multivariable logistic regression analysis of associations between HAZ, BAZ and pediatric kidney stones.

Variables	Model1		Model2		Model3	
	OR (95%CI)	*p*	OR (95%CI)	*p*	OR (95%CI)	*p*
BAZ	0.76 (0.74 ~ 0.78)	**<0.001**	0.79 (0.76 ~ 0.81)	**<0.001**	0.79 (0.76 ~ 0.81)	**<0.001**
*p*_for trend_	-	**<0.001**	-	**<0.001**	-	**<0.001**
HAZ	0.57 (0.55 ~ 0.59)	**<0.001**	0.64 (0.62 ~ 0.66)	**<0.001**	0.64 (0.61 ~ 0.66)	**<0.001**
*p*_for trend_	-	**<0.001**	-	**<0.001**	-	**<0.001**

Bold *p*-values indicate statistical significance (*p* < 0.05).

After adjusting for sex, ethnicity, and age (Model 2), the inverse association between BAZ and pediatric kidney stones remained significant (OR = 0.79, 95%CI: 0.76–0.81, *p* < 0.001, *p* for trend <0.001), and the inverse association of HAZ remained robust (OR = 0.64, 95%CI: 0.62–0.66, *p* < 0.001, *p* for trend <0.001).

With further adjustment for study center (Model 3), the association strengths remained essentially unchanged. Each 1-unit increase in BAZ was associated with 21% lower odds of kidney stones (OR = 0.79, 95%CI: 0.76–0.81, *p* < 0.001, *p* for trend <0.001); each 1-unit increase in HAZ was associated with 36% lower odds of kidney stones (OR = 0.64, 95%CI: 0.61–0.66, *p* < 0.001, *p* for trend <0.001). Across all models, the association strength between HAZ and pediatric kidney stones was consistently stronger than that of BAZ, suggesting that linear growth indicators may be more strongly associated with pediatric kidney stones.

In the joint model including both HAZ and BAZ simultaneously, both variables remained independently associated with pediatric urolithiasis. Each 1-unit increase in HAZ was associated with lower odds of urolithiasis (OR = 0.591, 95% CI: 0.569–0.612, *p* < 0.001), and each 1-unit increase in BAZ was also independently associated with lower odds of urolithiasis (OR = 0.703, 95% CI: 0.678–0.728, *p* < 0.001). The Pearson and Spearman correlation coefficients between HAZ and BAZ were −0.028 and 0.042, respectively, and the adjusted GVIF values for HAZ and BAZ were 1.049 and 1.057, indicating no meaningful multicollinearity (Supplementary Table S4).

In stratified analyses, the inverse associations of HAZ and BAZ were generally consistent across study centers and major ethnic subgroups (Supplementary Table S5). Center-by-ethnicity analyses showed similar patterns in strata with adequate case numbers, whereas several strata were not fitted because of sparse cases (Supplementary Table S6). Propensity score matching (1:1 nearest neighbor without replacement, exact on study center and ethnicity, caliper of 0.2 standard deviations of the logit of the propensity score) retained 2,178 case–control pairs. Center and ethnicity were exactly balanced after matching, and the largest standardized mean difference was 0.108 for sex, which was further controlled by covariate adjustment in the outcome model. In the matched sample, both HAZ (OR 0.668, 95% CI: 0.643–0.694, *p* < 0.001) and BAZ (OR 0.705, 95% CI: 0.674–0.737, *p* < 0.001) remained significantly inversely associated with urolithiasis. Consistent estimates were obtained with stabilized inverse probability weighting (HAZ OR 0.615, 95% CI: 0.578–0.656; BAZ OR 0.774, 95% CI: 0.726–0.826) and overlap weighting, which achieved near-perfect balance (maximum |SMD| < 0.001; HAZ OR 0.590, 95% CI: 0.567–0.615; BAZ OR 0.691, 95% CI: 0.666–0.718) (Supplementary Table S7; Supplementary Figures S2–S4).

Model diagnostics are summarized in Supplementary Table S13. The primary model (Model 3) showed good discrimination (AUC 0.834, 95% CI: 0.826–0.843) and excellent in-sample calibration (calibration intercept 0, calibration slope 1; Brier score 0.102). The Hosmer–Lemeshow test was statistically significant (*p* < 0.001); given the very large sample size, this test is sensitive to trivial deviations and is reported for reference only. With 2,180 events and 8 predictor parameters (events per variable 272.5), there was no meaningful risk of overfitting.

### Nonlinear association between HAZ, BAZ and pediatric kidney stones

Figure 2 illustrates the dose–response relationships between HAZ, BAZ and the odds of pediatric kidney stones using restricted cubic spline models. The RCS models used four knots located at the 5th, 35th, 65th, and 95th percentiles, and the exact knot locations are provided in Supplementary Table S8. After adjusting for sex, ethnicity, age, and study center, HAZ demonstrated a significant nonlinear inverse association with odds of kidney stones (*p* for overall <0.001, *p* for nonlinear <0.001; Figure 2A). When HAZ < 0, When HAZ < 0, the estimated odds rose steeply as HAZ decreased below 0; when HAZ ≥ 0, the decrease in estimated odds became more gradual and then plateaued at a relatively lower level.; when HAZ ≥ 0, the decrease in estimated odds became more gradual and then plateaued at a relatively lower level. This nonlinear pattern suggests that children with stunted growth (HAZ < -2) or poor growth have significantly higher odds of kidney stones. To further evaluate the clinical significance of growth stunting, we categorized HAZ according to WHO standards into non-stunting (HAZ ≥ -2), moderate stunting (−3 ≤ HAZ < -2), and severe stunting (HAZ < -3). Compared with non-stunted children, those with moderate stunting had nearly four-fold higher odds of kidney stones (adjusted OR = 3.98, 95% CI: 3.31–4.79, *p* < 0.001), while children with severe stunting exhibited even higher odds (adjusted OR = 4.29, 95% CI: 3.44–5.35, *p* < 0.001) after adjusting for center, gender, ethnicity, and age (Model 3). A significant dose–response relationship was observed across stunting categories (*p* for trend <0.001) (Supplementary Table S2). These findings indicate that growth stunting, particularly severe stunting, is strongly associated with higher odds of pediatric kidney stones and aligns with the nonlinear pattern observed in the restricted cubic spline analysis.

**Figure 2:**
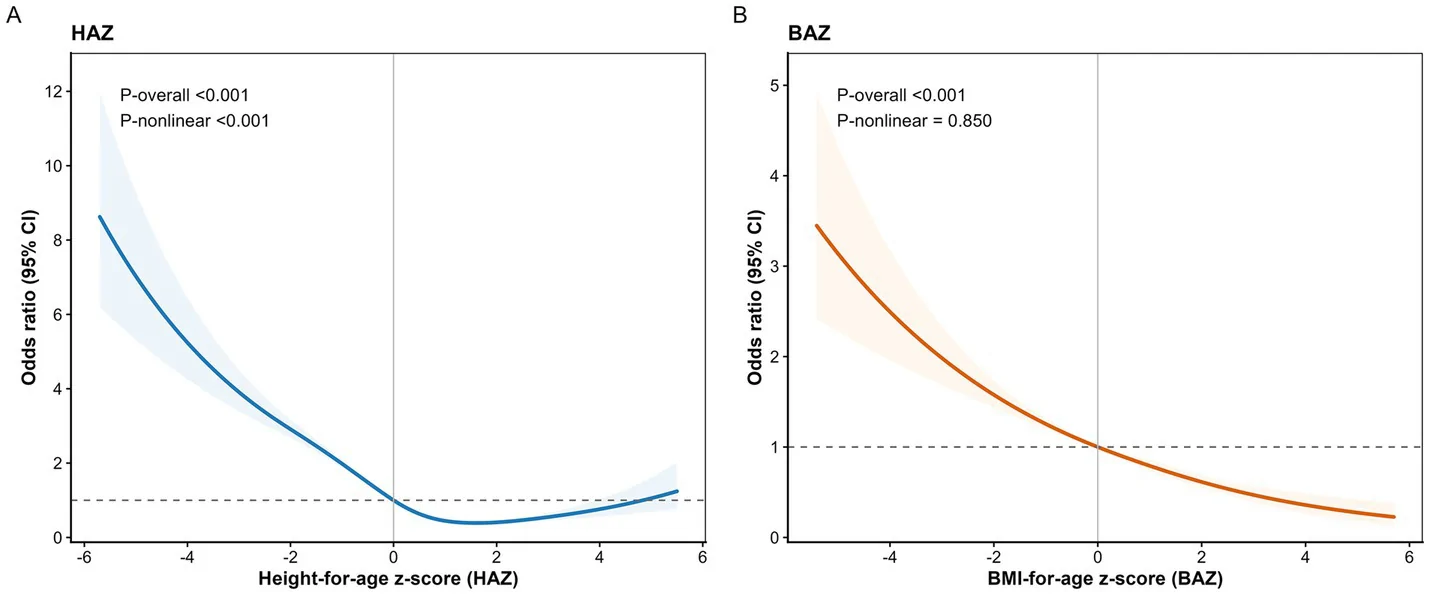
Restricted cubic spline analyses of the associations of HAZ and BAZ with pediatric urolithiasis. Four knots were placed at the 5th, 35th, 65th, and 95th percentiles. The knot locations were −2.360, −0.130, 0.930, and 2.647 for HAZ, and −2.180, −0.290, 0.860, and 2.940 for BAZ. Odds ratios were estimated using a *Z*-score of 0 as the reference. Shaded areas represent 95% confidence intervals. **(A)** HAZ showed a significant nonlinear inverse association. **(B)** BAZ showed a significant overall but approximately linear inverse association.

In contrast, although the overall association between BAZ and the odds of pediatric kidney stones was statistically significant (*p* for overall <0.001), it did not exhibit a significant nonlinear relationship (*p* for nonlinear = 0.850; Figure 2B; Supplementary Table S8). BAZ showed an approximately linear inverse association with odds of kidney stones, indicating that lower BAZ was associated with higher odds of kidney stones, but this association remained relatively uniform across the entire BAZ range without apparent inflection points or accelerated changes. In joint RCS sensitivity analyses, the overall inverse patterns remained consistent after mutual adjustment of HAZ and BAZ (Supplementary Figure S1; Supplementary Table S8).

Overall, the nonlinear association pattern of HAZ was more pronounced, particularly demonstrating a stronger association with kidney stone odds in the negative value range, while BAZ exhibited a relatively stable linear inverse association.

### Subgroup analyses of associations between HAZ, BAZ and pediatric kidney stones

To evaluate the robustness and heterogeneity of associations between HAZ, BAZ and pediatric kidney stones across different population characteristics, we conducted subgroup analyses stratified by study center, sex, ethnicity, age group, and nutritional status.

Subgroup analysis of HAZ (Figure 3) demonstrated that the inverse association between HAZ and pediatric kidney stones remained significant across all subgroups (all *p* < 0.001). However, the association strength varied significantly across subgroups. A significant interaction was observed between study centers (*p* for interaction <0.001), with center B showing a stronger association (OR = 0.56, 95%CI: 0.53–0.60) than center A (OR = 0.68, 95%CI: 0.65–0.71). Significant interaction was also found across age groups (*p* for interaction <0.001), and a relatively weaker inverse association in the <36 months group (OR = 0.76, 95%CI: 0.71–0.81). Stratification by BAZ revealed significant interaction (*p* for interaction <0.001), with stronger inverse associations between HAZ and kidney stone odds in normal-weight children (OR = 0.56, 95%CI: 0.53–0.59) and overweight/obese children (OR = 0.59, 95%CI: 0.56–0.63) compared to thin children (OR = 0.82, 95%CI: 0.75–0.90). No significant interactions were found for sex (*p* for interaction = 0.108) or ethnicity (*p* for interaction = 0.058), suggesting relatively consistent inverse associations of HAZ with kidney stone odds across different sexes and ethnicities. After correction for multiple testing, the interactions of HAZ with study center, age group, and BAZ-defined nutritional status remained significant, whereas interactions with sex and ethnicity were not significant (Supplementary Tables S9, S11).

**Figure 3:**
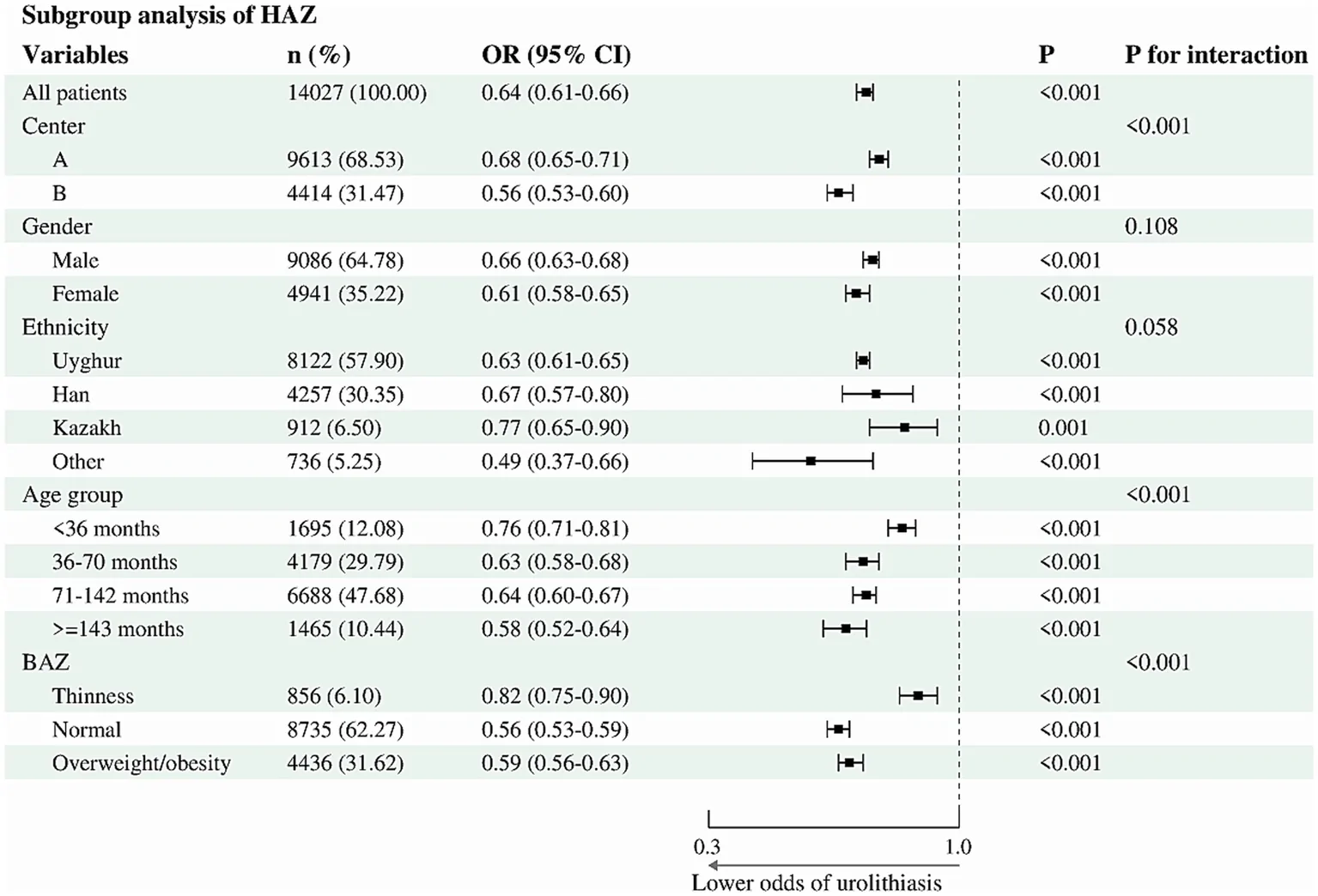
Subgroup analysis of the association between HAZ and pediatric kidney stones. Associations were stratified by study center, sex, ethnicity, age group, and nutritional status. The strength of HAZ’s inverse association varied across subgroups, with interaction effects noted for study center, age, and BAZ category. BAZ, BMI-for-age *Z*-score; CI, confidence interval; HAZ, height-for-age *Z*-score; OR, odds ratio.

Subgroup analysis of BAZ (Figure 4) similarly showed that the inverse association between BAZ and pediatric kidney stones remained significant in nearly all subgroups. The interaction between BAZ and age group was nominally significant (*p* for interaction = 0.020) and remained borderline after FDR correction (FDR-adjusted *p* = 0.049), but it did not remain significant after Bonferroni correction (Bonferroni-adjusted *p* = 0.197); therefore, this result should be interpreted cautiously (Supplementary Tables S10, S11). Stratification by HAZ showed no significant interaction (*p* for interaction = 0.559), with stable inverse associations of BAZ in children with moderate stunting (OR = 0.70, 95%CI: 0.61–0.79), severe stunting (OR = 0.76, 95%CI: 0.67–0.87), and non-stunting (OR = 0.74, 95%CI: 0.71–0.77). No significant interactions were found for study center (*p* for interaction = 0.179), sex (*p* for interaction = 0.753), or ethnicity (*p* for interaction = 0.617), indicating good consistency of BAZ’s inverse association across different centers, sexes, and ethnicities.

**Figure 4:**
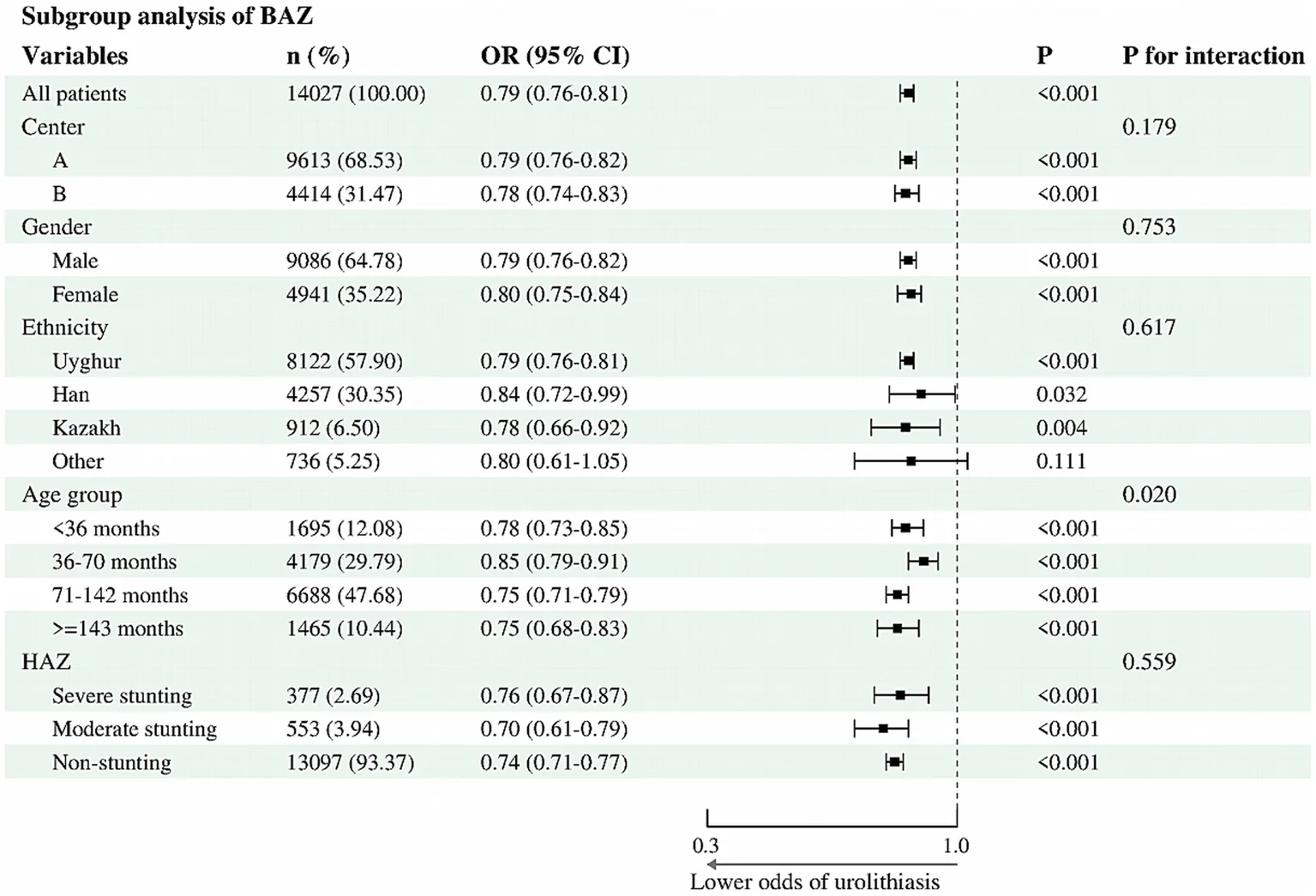
Subgroup analysis of the association between BAZ and pediatric kidney stones. Associations remained significant across most subgroups, with minor variation by age group. No significant interaction was observed for study center, sex, or ethnicity. BAZ, BMI-for-age *Z*-score; CI, confidence interval; OR, odds ratio.

Sensitivity analyses using mixed-effects logistic regression with random intercepts for center and calendar year yielded materially unchanged fixed-effect estimates (HAZ OR 0.65, 95% CI: 0.62–0.68; BAZ OR 0.79, 95% CI: 0.76–0.82). The variance partition coefficient for center was small (3.7%), indicating limited center-level clustering and supporting the appropriateness of the primary fixed-effects models.

Overall, the inverse associations between HAZ, BAZ and pediatric kidney stones remained robust across most subgroups, but the association strength of HAZ was more notably influenced by study center, age, and nutritional status, while BAZ demonstrated stronger cross-subgroup consistency. After adjusting for potential confounders, thinness (BAZ < −2) was associated with increased odds of urolithiasis (adjusted OR: 1.86, 95% CI: 1.57–2.19), while overweight and obesity showed lower odds (Supplementary Table S3).

## Discussion

This large-scale multicenter study of 14,027 children in Xinjiang, China, systematically evaluated the associations between growth indicators (HAZ and BAZ) and pediatric kidney stones. Our principal findings demonstrate that both HAZ and BAZ were inversely associated with the odds of kidney stones, with HAZ exhibiting a stronger and nonlinear relationship. These associations remained robust after adjusting for demographic and clinical confounders and were consistent across most subgroups, though with notable heterogeneity by age, study center, and nutritional status.

The inverse association between HAZ and pediatric kidney stones observed in our study aligns with emerging evidence linking growth impairment to metabolic disorders (21). Children with lower HAZ had substantially higher odds of kidney stones, with the association being particularly pronounced when HAZ fell below zero. This nonlinear pattern suggests a threshold effect, whereby growth faltering may trigger metabolic derangements conducive to stone formation. Previous studies have established that chronic malnutrition impairs renal tubular function and alters urinary biochemistry, including reduced citrate excretion and increased urinary calcium-to-creatinine ratios, both established lithogenic factors (22, 23). Our findings extend this literature by demonstrating that HAZ, as a comprehensive growth indicator, may serve as a practical screening tool for identifying high-risk children in resource-limited settings where detailed metabolic evaluations are unavailable. The categorical analysis based on WHO growth standards provided additional clinical insights (Supplementary Table S2). Children with moderate and severe stunting demonstrated 3.98-fold and 4.29-fold increased odds of kidney stones, respectively, compared with non-stunted children. This stepwise risk elevation underscores the importance of routine HAZ screening in pediatric primary care, particularly in regions with high stunting prevalence, as early identification of growth-impaired children may facilitate earlier preventive intervention.

The inverse association of higher BAZ with lower odds of pediatric kidney stones observed in our study contrasts with evidence from adult cohorts, in which higher BMI or obesity has been associated with higher odds of kidney stones or more lithogenic urinary profiles (24). However, pediatric findings remain heterogeneous, suggesting that the relationship between body size and stone risk may differ by age, nutritional background, socioeconomic context, and metabolic phenotype (25). This apparent paradox likely reflects the distinct nutritional context of our study population. In Xinjiang, lower BAZ predominantly indicates undernutrition rather than healthy weight, whereas higher BAZ may reflect adequate nutritional status rather than obesity. Indeed, only 31.62% of our cohort was classified as overweight/obese, and the median BAZ in the stone group was negative (−0.25), suggesting prevalent undernutrition. Dietary composition and fluid intake, rather than adiposity itself, may also underlie the association: in this setting, children with higher BAZ may have better overall dietary quality and hydration, both of which are associated with lower stone risk; because dietary intake and hydration behaviors were not captured in this retrospective dataset, these explanations could not be formally tested and are offered as hypotheses for prospective studies. This interpretation is supported by studies from other developing regions showing that malnutrition-related metabolic acidosis and micronutrient deficiencies promote urolithiasis (26). The linear association between BAZ and stone risk, without significant nonlinearity, suggests that higher BAZ, reflecting nutritional adequacy in this population, was consistently associated with lower odds of urolithiasis across the BAZ spectrum in this population. Notably, the inverse association between BAZ and pediatric kidney stones observed here contrasts with the well-documented positive association between obesity and stone risk in adult populations from high-income settings. In our cohort, lower BAZ predominantly reflected undernutrition rather than healthy weight, highlighting the importance of nutritional context in interpreting body size–stone associations.

Several biological mechanisms may underlie the observed associations. First, chronic malnutrition, reflected by low HAZ, induces metabolic acidosis through inadequate dietary alkali intake and impaired renal acid excretion (27). Chronic metabolic acidosis reduces urinary citrate excretion, a key inhibitor of calcium stone formation, and increases urinary calcium excretion through bone buffering. Second, micronutrient deficiencies common in malnourished children—particularly magnesium, potassium, and vitamin B6—impair stone inhibitor synthesis and promote crystallization (17, 28, 29). The lower serum magnesium and potassium levels observed in our stone group are consistent with this pathway. Third, growth impairment may reflect chronic inflammation, evidenced by elevated platelet counts and fibrinogen levels in the stone group, which has been implicated in renal tubular injury and altered stone matrix protein expression (30).

The stronger association of HAZ compared to BAZ may reflect HAZ’s capture of cumulative nutritional insults over time, whereas BAZ represents more recent nutritional status. Linear growth is particularly sensitive to chronic deficiencies in protein, zinc, and vitamin A, which also influence renal tubular function and urinary biochemistry (31, 32). Additionally, the nonlinear HAZ-stone relationship suggests that severe growth faltering triggers qualitatively different metabolic derangements than mild growth impairment, possibly involving hormonal dysregulation (e.g., growth hormone-IGF-1 axis) (33, 34).

The significant heterogeneity observed across subgroups provides important insights for targeted interventions. The stronger HAZ-stone association in older children (≥143 months) and center B may reflect cumulative exposure to nutritional deficits or regional differences in dietary patterns and water quality. The Uyghur ethnicity’s disproportionate stone burden (92.61% of cases) likely reflects both genetic susceptibility and cultural dietary practices, including high salt intake and low fluid consumption (20, 35). Notably, the HAZ-stone association was attenuated in thin children (BAZ < -2), suggesting that acute malnutrition may obscure the effects of chronic growth impairment, or that these children represent a distinct phenotype requiring different preventive strategies.

These findings should be contextualized within the existing evidence base. In high-income settings, where calcium oxalate stones and obesity-related metabolic risk predominate, higher BMI is typically associated with higher stone risk; our inverse BAZ association should therefore not be extrapolated to those settings. In endemic regions such as Xinjiang, however, where undernutrition remains prevalent, our findings raise the hypothesis that growth monitoring programs could be leveraged for kidney stone risk stratification. Whether children with HAZ < -2 (moderate-to-severe stunting) might benefit from enhanced surveillance—such as urinalysis screening and dietary counseling focused on adequate hydration, citrate-rich foods, and sodium restriction—is a hypothesis that requires confirmation in prospective studies before any clinical recommendation can be made. In resource-constrained settings like Xinjiang, where routine metabolic stone evaluations are impractical, HAZ measurement offers a simple, cost-effective risk assessment tool. Interventions addressing childhood malnutrition—such as micronutrient supplementation, dietary diversification, and treatment of underlying infections—may offer dual potential benefits for growth and stone prevention, although this hypothesis requires confirmation in prospective studies.

This study’s strengths include its large sample size, multicenter design, comprehensive covariate adjustment, and rigorous statistical approaches including nonlinear modeling and extensive subgroup analyses. The use of standardized WHO growth indicators enhances generalizability and comparability with international studies. However, several limitations should be acknowledged. First, because this was a retrospective case–control study, the temporal relationship between growth indicators and urolithiasis cannot be firmly established, and reverse causality remains possible, particularly if chronic or recurrent stone disease affected growth trajectories. Second, although HAZ and BAZ were calculated using age- and sex-specific WHO references and all primary models adjusted for sex and exact age in months, direct information on pubertal status or biological maturation, such as Tanner stage or age at menarche, was not available; therefore, residual confounding related to pubertal timing may remain, especially in older children. Third, we lacked detailed information on dietary intake, fluid consumption, socioeconomic status, rural/urban residence, ambient temperature, and stone composition, which limited our ability to assess lifestyle, environmental, and stone-type-specific mechanisms. Fourth, urinalysis was based on first-morning urine samples collected on the second day of hospitalization or health examination. Although this approach reduced random variation related to daytime fluid intake, information on intravenous hydration or emergency treatment before urine collection was not consistently available. Thus, the higher rate of proteinuria and lower urine specific gravity among cases may partly reflect acute illness, urinary tract irritation, hematuria, infection, obstruction, or pre-sampling hydration. Fifth, cases were identified among hospitalized children whereas controls were recruited from routine health examinations; this asymmetry in source populations may introduce selection bias related to healthcare-seeking behavior, and measurements in cases were obtained at admission, potentially during acute illness, fasting, or dehydration. The lower hemoglobin and albumin levels and elevated inflammatory markers observed among cases may therefore partly reflect acute-illness physiology rather than chronic nutritional status; for this reason, laboratory parameters were reported descriptively and were not included in the etiologic models. Sixth, the case group was predominantly Uyghur (92.61%), with only 72 Han Chinese cases; subgroup estimates for Han and other smaller ethnic groups were therefore imprecise, and generalizability of our findings to ethnically diverse populations is limited. Seventh, the study was conducted in a region with a uniquely elevated burden of pediatric urolithiasis, shaped by local environmental factors such as water mineral content, ambient temperature, and dietary patterns; these factors were not directly measured, and seasonal variation in stone presentation could not be captured. Our findings may therefore not extrapolate to settings with different stone epidemiology—particularly high-income settings in which calcium oxalate stones and obesity-related metabolic risk predominate. Future prospective studies with longitudinal growth assessment, direct pubertal evaluation, detailed environmental and dietary data, urinary biochemistry, and stone composition analysis are needed to confirm these findings and clarify the underlying mechanisms.

## Conclusion

In this large multicenter study, lower HAZ and BAZ were independently associated with higher odds of pediatric urolithiasis, with HAZ demonstrating a stronger, nonlinear relationship. These findings suggest that growth monitoring may facilitate early identification of high-risk children, and that interventions addressing childhood malnutrition may have implications for urolithiasis prevention in endemic regions, although these associations and any growth-targeted preventive strategies require confirmation in prospective studies.

## Data Availability

The raw data supporting the conclusions of this article will be made available by the authors, without undue reservation.
