# Supplementary material for: Associations of height-for-age and BMI-for-age Z-scores with pediatric urolithiasis: a multicenter study
Source: Front Nutr. 2026 Sep 28;13:1880262. doi: 10.3389/fnut.2026.1880262 (PMC13656848; doi:10.3389/fnut.2026.1880262)
Supplement: Supplementary file 1 [file Supplementary_file_1.DOCX]

**Supplementary Tables**

**Table S1. Comparison of Baseline Characteristics and Clinical Parameters Between Groups**

| Variable | Total  (n = 14027) | Center A (n = 9613) | Center B (n = 4414) | Statistic | *P* | SMD |
| --- | --- | --- | --- | --- | --- | --- |
| Result, n (%) |  |  |  | χ²=1.703 | 0.192 |  |
| Non-Stone | 11847 (84.46) | 8145 (84.73) | 3702 (83.87) |  |  | -0.023 |
| Stone | 2180 (15.54) | 1468 (15.27) | 712 (16.13) |  |  | 0.023 |
| Gender, n (%) |  |  |  | χ²=0.319 | 0.572 |  |
| Male | 9086 (64.78) | 6212 (64.62) | 2874 (65.11) |  |  | 0.010 |
| Female | 4941 (35.22) | 3401 (35.38) | 1540 (34.89) |  |  | -0.010 |
| Ethnicity, n (%) |  |  |  | χ²=26.610 | <.001 |  |
| Uyghur | 8122 (57.9) | 5451 (56.70) | 2671 (60.51) |  |  | 0.078 |
| Han | 4257 (30.35) | 3020 (31.42) | 1237 (28.02) |  |  | -0.076 |
| Kazakh | 912 (6.5) | 657 (6.83) | 255 (5.78) |  |  | -0.045 |
| Other | 736 (5.25) | 485 (5.05) | 251 (5.69) |  |  | 0.028 |
| Age (Month) | 80.00 (53.00, 114.00) | 71.00 (47.00, 105.00) | 97.00 (70.00, 127.00) | Z=-29.195 | <.001 | 0.561 |
| Haz | 0.40 (-0.54, 1.30) | 0.34 (-0.62, 1.23) | 0.54 (-0.40, 1.45) | Z=-8.119 | <.001 | 0.128 |
| Baz | 0.28 (-0.68, 1.34) | 0.30 (-0.66, 1.34) | 0.24 (-0.74, 1.35) | Z=-1.705 | 0.088 | -0.042 |
| Rbc | 4.74 (4.49, 5.00) | 4.74 (4.49, 5.00) | 4.74 (4.49, 4.99) | Z=-0.100 | 0.920 | 0.017 |
| Mcv | 82.40 (79.40, 85.30) | 82.10 (78.90, 85.00) | 83.20 (80.40, 85.70) | Z=-13.139 | <.001 | 0.255 |
| Mchc | 329.00 (320.00, 338.00) | 330.00 (320.00, 339.00) | 328.00 (321.00, 336.00) | Z=-6.001 | <.001 | -0.057 |
| Mch | 27.20 (26.10, 28.20) | 27.10 (26.00, 28.20) | 27.40 (26.30, 28.30) | Z=-7.833 | <.001 | 0.177 |
| Rdw Cv | 13.20 (12.60, 13.80) | 13.20 (12.60, 13.80) | 13.20 (12.70, 13.70) | Z=-0.495 | 0.620 | -0.090 |
| Hct | 0.39 (0.37, 0.42) | 0.39 (0.37, 0.42) | 0.40 (0.37, 0.42) | Z=-0.600 | 0.548 | -0.810 |
| Hb | 128.00 (121.00, 135.00) | 128.00 (120.00, 135.00) | 129.00 (122.00, 136.00) | Z=-7.375 | <.001 | 0.091 |
| Plt | 331.00 (282.00, 388.00) | 332.00 (283.00, 390.00) | 330.00 (282.00, 382.00) | Z=-2.359 | 0.018 | -0.065 |
| Mpv | 10.10 (9.20, 16.90) | 10.60 (9.40, 20.50) | 9.50 (8.90, 10.40) | Z=-34.375 | <.001 | -0.707 |
| Pdw | 11.70 (10.10, 15.30) | 11.40 (10.10, 14.40) | 14.30 (10.00, 15.70) | Z=-17.567 | <.001 | 0.320 |
| Plb | 0.32 (0.27, 0.37) | 0.32 (0.28, 0.37) | 0.31 (0.27, 0.35) | Z=-9.628 | <.001 | -0.195 |
| Sd | 39.40 (37.70, 41.40) | 39.20 (37.50, 41.20) | 39.80 (38.20, 41.70) | Z=-12.286 | <.001 | 0.178 |
| U Tp, n (%) |  |  |  | χ²=6.533 | 0.011 |  |
| Positive | 1259 (8.98) | 903 (9.39) | 356 (8.07) |  |  | -0.049 |
| Negative | 12768 (91.02) | 8710 (90.61) | 4058 (91.93) |  |  | 0.049 |
| Sis, n (%) |  |  |  | χ²=57.374 | <.001 |  |
| 0 | 11305 (80.59) | 7585 (78.90) | 3720 (84.28) |  |  | 0.148 |
| 1 | 2654 (18.92) | 1982 (20.62) | 672 (15.22) |  |  | -0.150 |
| 2 | 68 (0.48) | 46 (0.48) | 22 (0.50) |  |  | 0.003 |
| Uro, n (%) |  |  |  | χ²=14.660 | <.001 |  |
| Positive | 408 (2.91) | 315 (3.28) | 93 (2.11) |  |  | -0.081 |
| Negative | 13619 (97.09) | 9298 (96.72) | 4321 (97.89) |  |  | 0.081 |
| Bil, n (%) |  |  |  | χ²=0.021 | 0.886 |  |
| Positive | 200 (1.43) | 138 (1.44) | 62 (1.40) |  |  | -0.003 |
| Negative | 13827 (98.57) | 9475 (98.56) | 4352 (98.60) |  |  | 0.003 |
| Glu, n (%) |  |  |  | χ²=44.910 | <.001 |  |
| Positive | 70 (0.5) | 22 (0.23) | 48 (1.09) |  |  | 0.083 |
| Negative | 13957 (99.5) | 9591 (99.77) | 4366 (98.91) |  |  | -0.083 |
| Ket, n (%) |  |  |  | χ²=1.744 | 0.187 |  |
| Positive | 444 (3.17) | 317 (3.30) | 127 (2.88) |  |  | -0.025 |
| Negative | 13583 (96.83) | 9296 (96.70) | 4287 (97.12) |  |  | 0.025 |
| Sg | 1.02 (1.02, 1.03) | 1.02 (1.02, 1.03) | 1.02 (1.02, 1.02) | Z=-11.411 | <.001 | 0.001 |
| Ph | 5.88 (5.50, 6.00) | 5.88 (5.50, 6.00) | 5.89 (5.72, 6.00) | Z=-5.156 | <.001 | 0.111 |
| Alt | 14.00 (11.00, 19.00) | 14.00 (11.00, 18.00) | 15.20 (12.00, 20.00) | Z=-13.065 | <.001 | 0.070 |
| Ast | 25.00 (21.60, 30.00) | 25.00 (21.00, 30.00) | 26.00 (22.00, 30.00) | Z=-4.273 | <.001 | -0.018 |
| Ggt | 12.00 (10.00, 14.00) | 12.00 (10.00, 14.00) | 13.00 (11.00, 15.00) | Z=-16.505 | <.001 | 0.059 |
| Dbil | 2.40 (1.80, 3.20) | 2.40 (1.80, 3.30) | 2.35 (1.80, 3.10) | Z=-2.929 | 0.003 | -0.055 |
| Tbil | 6.80 (5.20, 9.30) | 6.60 (5.18, 8.80) | 7.30 (5.20, 10.21) | Z=-9.830 | <.001 | 0.091 |
| Alb | 43.00 (40.89, 45.10) | 42.63 (40.60, 44.72) | 43.80 (41.50, 45.80) | Z=-17.648 | <.001 | 0.284 |
| Ibil | 4.21 (2.89, 6.00) | 4.10 (2.85, 5.70) | 4.53 (2.90, 6.83) | Z=-9.577 | <.001 | 0.099 |
| Tp | 68.90 (65.40, 72.60) | 68.40 (65.10, 71.70) | 70.37 (66.08, 74.30) | Z=-18.315 | <.001 | 0.145 |
| Glo | 25.30 (21.66, 28.40) | 24.70 (20.60, 27.70) | 26.80 (23.71, 29.60) | Z=-25.694 | <.001 | 0.257 |
| Crea | 35.00 (29.00, 41.50) | 34.00 (28.20, 40.70) | 36.60 (31.00, 43.00) | Z=-14.791 | <.001 | 0.041 |
| Bun | 4.40 (3.62, 5.24) | 4.36 (3.60, 5.22) | 4.44 (3.68, 5.26) | Z=-3.783 | <.001 | 0.021 |
| Ua | 240.26 (200.96, 288.66) | 236.77 (197.80, 284.00) | 247.45 (208.00, 299.61) | Z=-9.487 | <.001 | 0.087 |
| K | 4.32 (4.10, 4.56) | 4.30 (4.08, 4.54) | 4.35 (4.14, 4.59) | Z=-6.816 | <.001 | 0.044 |
| Cl | 105.00 (103.20, 107.00) | 105.00 (103.31, 107.00) | 105.00 (103.10, 106.55) | Z=-7.238 | <.001 | -0.124 |
| Na | 139.40 (138.00, 141.00) | 139.00 (138.00, 141.00) | 139.76 (138.30, 141.00) | Z=-9.336 | <.001 | 0.077 |
| Mg2 | 0.87 (0.82, 0.92) | 0.88 (0.83, 0.92) | 0.84 (0.80, 0.90) | Z=-20.675 | <.001 | 0.007 |
| Ca | 2.40 (2.33, 2.47) | 2.40 (2.34, 2.47) | 2.41 (2.33, 2.48) | Z=-0.144 | 0.886 | -0.010 |
| Che | 9.00 (8.00, 10.00) | 9.16 (8.10, 10.28) | 8.76 (7.83, 9.51) | Z=-17.781 | <.001 | -0.362 |
| Pt Inr | 1.03 (0.98, 1.08) | 1.02 (0.97, 1.07) | 1.05 (1.00, 1.10) | Z=-21.176 | <.001 | 0.280 |
| Pt | 11.90 (11.30, 12.80) | 12.00 (11.30, 12.90) | 11.80 (11.30, 12.50) | Z=-7.638 | <.001 | -0.123 |
| Ptr | 1.04 (0.98, 1.11) | 1.04 (0.98, 1.12) | 1.03 (0.98, 1.10) | Z=-3.756 | <.001 | -0.095 |
| Pta | 94.00 (86.00, 102.80) | 95.00 (86.30, 103.60) | 92.60 (85.00, 101.00) | Z=-8.677 | <.001 | -0.169 |
| Tt | 17.40 (16.14, 18.59) | 17.80 (16.80, 18.80) | 16.10 (15.00, 17.60) | Z=-6.814 | <.001 | -0.197 |
| Aptt | 31.00 (28.50, 35.50) | 30.80 (28.20, 36.00) | 31.40 (28.90, 34.90) | Z=-2.499 | 0.012 | -0.045 |
| Fbg | 2.47 (2.14, 2.89) | 2.41 (2.09, 2.84) | 2.58 (2.25, 3.00) | Z=-16.189 | <.001 | 0.237 |
| D Dimer | 0.23 (0.15, 0.35) | 0.24 (0.17, 0.38) | 0.20 (0.13, 0.31) | Z=-17.220 | <.001 | -0.152 |

Table footnote: Continuous variables are presented as median (interquartile range), and categorical variables as number (percentage). Between-group comparisons were performed using Mann-Whitney U test (continuous variables) or chi-square test (categorical variables). SMD: Standardized Mean Difference. Abbreviations: HAZ, height-for-age Z-score; BAZ, BMI-for-age Z-score; RBC, red blood cell count; MCV, mean corpuscular volume; MCHC, mean corpuscular hemoglobin concentration; MCH, mean corpuscular hemoglobin; RDW-CV, red cell distribution width-coefficient of variation; HCT, hematocrit; Hb, hemoglobin; PLT, platelet count; MPV, mean platelet volume; PDW, platelet distribution width; U-TP, urinary protein; SG, specific gravity; ALT, alanine aminotransferase; AST, aspartate aminotransferase; GGT, gamma-glutamyl transferase; DBIL, direct bilirubin; TBIL, total bilirubin; ALB, albumin; IBIL, indirect bilirubin; TP, total protein; GLO, globulin; CREA, creatinine; BUN, blood urea nitrogen; UA, uric acid; alkaline phosphatase; CHE, cholinesterase; PT, prothrombin time; APTT, activated partial thromboplastin time; FBG, fibrinogen.

**Table S2. Association between HAZ categories and pediatric urolithiasis**

| Variables | Model 1 | | Model 2 | | Model 3 | |
| --- | --- | --- | --- | --- | --- | --- |
|  | OR (95% CI) | *P* | OR (95% CI) | *P* | OR (95% CI) | *P* |
| HAZ >= -2 (non-stunting) | 1.00 (Reference) |  | 1.00 (Reference) |  | 1.00 (Reference) |  |
| -3 <= HAZ < -2 (moderate stunting) | 5.60 (4.70 ~ 6.66) | <0.001 | 3.97 (3.30 ~ 4.78) | <0.001 | 3.98 (3.31 ~ 4.79) | <0.001 |
| HAZ < -3 (severe stunting) | 6.51 (5.28 ~ 8.01) | <0.001 | 4.29 (3.44 ~ 5.34) | <0.001 | 4.29 (3.44 ~ 5.35) | <0.001 |
| *P* for trend |  | <0.001 |  | <0.001 |  | <0.001 |

HAZ, height-for-age z-score; OR, odds ratio; CI, confidence interval.

**Table S3. Association between BAZ categories and pediatric urolithiasis**

| Variables | Model 1 | | Model 2 | | Model 3 | |
| --- | --- | --- | --- | --- | --- | --- |
|  | OR (95% CI) | *P* | OR (95% CI) | *P* | OR (95% CI) | *P* |
| -2 <= BAZ <= 1 (normal) | 1.00 (Reference) |  | 1.00 (Reference) |  | 1.00 (Reference) |  |
| BAZ < -2 (thinness) | 2.10 (1.80 ~ 2.46) | <0.001 | 1.87 (1.58 ~ 2.21) | <0.001 | 1.86 (1.57 ~ 2.19) | <0.001 |
| 1 < BAZ <= 2 (overweight) | 0.64 (0.56 ~ 0.73) | <0.001 | 0.67 (0.58 ~ 0.77) | <0.001 | 0.66 (0.58 ~ 0.77) | <0.001 |
| BAZ > 2 (obesity) | 0.39 (0.33 ~ 0.47) | <0.001 | 0.45 (0.37 ~ 0.54) | <0.001 | 0.45 (0.37 ~ 0.54) | <0.001 |
| *P* for trend |  | <0.001 |  | <0.001 |  | <0.001 |

BAZ, body mass index-for-age z-score; OR, odds ratio; CI, confidence interval.

Model 1: crude model.

Model 2: adjusted for gender, ethnicity, and age_month.

Model 3: adjusted for center, gender, ethnicity, and age_month.

P for trend was calculated by modeling ordered categories as a continuous term in logistic regression models.

**Table S4. Joint HAZ/BAZ models and multicollinearity diagnostics.**

*A. Logistic regression models. Models were adjusted for sex, ethnicity, age, and study center where applicable.*

| Model | Exposure | OR (95% CI) | *P* |
| --- | --- | --- | --- |
| HAZ only + covariates | HAZ | 0.636 (0.614, 0.658) | <0.001 |
| BAZ only + covariates | BAZ | 0.789 (0.764, 0.815) | <0.001 |
| HAZ + BAZ jointly + covariates | HAZ | 0.591 (0.569, 0.612) | <0.001 |
| HAZ + BAZ jointly + covariates | BAZ | 0.703 (0.678, 0.728) | <0.001 |

*B. Correlation between HAZ and BAZ.*

| Method | Correlation coefficient | *P* |
| --- | --- | --- |
| Pearson | -0.028 | 0.001 |
| Spearman | 0.042 | <0.001 |

*C. Variance inflation diagnostics from the joint multivariable model. For categorical variables, adjusted GVIF = GVIF^(1/(2*Df)).*

| Variable | GVIF | Df | Adjusted GVIF |
| --- | --- | --- | --- |
| haz | 1.100 | 1 | 1.049 |
| baz | 1.118 | 1 | 1.057 |
| gender | 1.015 | 1 | 1.007 |
| ethnicity | 1.018 | 3 | 1.003 |
| age_month | 1.123 | 1 | 1.060 |
| center | 1.089 | 1 | 1.043 |

**Table S5. Center-specific and ethnicity-specific joint logistic regression models.**

*Each model included both HAZ and BAZ simultaneously. Center-specific models were adjusted for sex, ethnicity, and age; ethnicity-specific models were adjusted for sex, study center, and age.*

| Stratification | Level | Exposure | OR (95% CI) | *P* | n | Cases | Controls |
| --- | --- | --- | --- | --- | --- | --- | --- |
| Center | Center A | HAZ | 0.622 (0.596, 0.650) | <0.001 | 9613 | 1468 | 8145 |
| Center | Center A | BAZ | 0.702 (0.672, 0.733) | <0.001 | 9613 | 1468 | 8145 |
| Center | Center B | HAZ | 0.532 (0.497, 0.569) | <0.001 | 4414 | 712 | 3702 |
| Center | Center B | BAZ | 0.703 (0.658, 0.749) | <0.001 | 4414 | 712 | 3702 |
| Ethnicity | Uyghur | HAZ | 0.580 (0.558, 0.603) | <0.001 | 8122 | 2019 | 6103 |
| Ethnicity | Uyghur | BAZ | 0.689 (0.663, 0.716) | <0.001 | 8122 | 2019 | 6103 |
| Ethnicity | Han | HAZ | 0.662 (0.557, 0.793) | <0.001 | 4257 | 72 | 4185 |
| Ethnicity | Han | BAZ | 0.837 (0.716, 0.973) | 0.022 | 4257 | 72 | 4185 |
| Ethnicity | Kazakh | HAZ | 0.711 (0.597, 0.844) | <0.001 | 912 | 66 | 846 |
| Ethnicity | Kazakh | BAZ | 0.719 (0.598, 0.860) | <0.001 | 912 | 66 | 846 |
| Ethnicity | Other | HAZ | 0.488 (0.359, 0.657) | <0.001 | 736 | 23 | 713 |
| Ethnicity | Other | BAZ | 0.823 (0.618, 1.081) | 0.170 | 736 | 23 | 713 |

**Table S6. Center-by-ethnicity stratified joint logistic regression models.**

*Each fitted model included both HAZ and BAZ simultaneously and was adjusted for sex and age. Strata with fewer than 20 cases or controls were not fitted.*

| Center / ethnicity | Exposure | OR (95% CI) | *P* | n | Cases | Controls | Note |
| --- | --- | --- | --- | --- | --- | --- | --- |
| Center A / Uyghur | HAZ | 0.608 (0.580, 0.637) | <0.001 | 5451 | 1332 | 4119 |  |
| Center A / Uyghur | BAZ | 0.686 (0.654, 0.719) | <0.001 | 5451 | 1332 | 4119 |  |
| Center B / Uyghur | HAZ | 0.536 (0.499, 0.573) | <0.001 | 2671 | 687 | 1984 |  |
| Center B / Uyghur | BAZ | 0.696 (0.650, 0.743) | <0.001 | 2671 | 687 | 1984 |  |
| Center A / Han | HAZ | 0.685 (0.570, 0.827) | <0.001 | 3020 | 66 | 2954 |  |
| Center A / Han | BAZ | 0.823 (0.699, 0.964) | 0.017 | 3020 | 66 | 2954 |  |
| Center B / Han | HAZ |  |  | 1237 | 6 | 1231 | Not fitted: fewer than 20 cases or controls in this stratum |
| Center B / Han | BAZ |  |  | 1237 | 6 | 1231 | Not fitted: fewer than 20 cases or controls in this stratum |
| Center A / Kazakh | HAZ | 0.791 (0.650, 0.959) | 0.017 | 657 | 50 | 607 |  |
| Center A / Kazakh | BAZ | 0.722 (0.586, 0.884) | 0.002 | 657 | 50 | 607 |  |
| Center B / Kazakh | HAZ |  |  | 255 | 16 | 239 | Not fitted: fewer than 20 cases or controls in this stratum |
| Center B / Kazakh | BAZ |  |  | 255 | 16 | 239 | Not fitted: fewer than 20 cases or controls in this stratum |
| Center A / Other | HAZ | 0.525 (0.368, 0.742) | <0.001 | 485 | 20 | 465 |  |
| Center A / Other | BAZ | 0.822 (0.604, 1.101) | 0.200 | 485 | 20 | 465 |  |
| Center B / Other | HAZ |  |  | 251 | 3 | 248 | Not fitted: fewer than 20 cases or controls in this stratum |
| Center B / Other | BAZ |  |  | 251 | 3 | 248 | Not fitted: fewer than 20 cases or controls in this stratum |

**Table S7. Propensity score matched and propensity score weighted sensitivity analyses.**

*The propensity score was estimated by logistic regression (generalized linear model, logit link) with study center, ethnicity, sex, and age (months) as covariates. HAZ and BAZ were not included in the propensity score model because they were the primary exposures. All outcome models included HAZ and BAZ simultaneously and were adjusted for study center, ethnicity, sex, and age (months).*

**A. Propensity score methods and diagnostics**

| Item | Specification |
| --- | --- |
| Propensity score model | Logistic regression with study center, ethnicity, sex, and age (months) as covariates; HAZ and BAZ were not included because they were the primary exposures. |
| Matching algorithm | Nearest-neighbor matching, 1:1, without replacement, with exact matching on study center and ethnicity. |
| Distance measure | Propensity score estimated by logistic regression. |
| Caliper | 0.2 standard deviations of the propensity score distance (std.caliper = TRUE). |
| Matched sample size | 2,178 case–control pairs (2,178 cases and 2,178 controls); 2 cases (0.09%) remained unmatched; no observations were discarded by the caliper. |
| Outcome model in matched sample | Joint logistic regression adjusted for study center, ethnicity, sex, and age; standard errors computed using a survey design clustered on matched pairs. |
| Inverse probability of treatment weighting (IPTW) | Stabilized weights targeting the average treatment effect (ATE). |
| Overlap weighting | Weights targeting the average treatment effect in the overlap population (ATO): cases weighted by 1 − PS, controls weighted by PS. |
| Weighted outcome models | Joint logistic regression with robust (sandwich) standard errors, adjusted for study center, ethnicity, sex, and age. |
| Balance assessment | Standardized mean differences (SMDs) computed with the cobalt package; \|SMD\| < 0.1 was considered acceptable balance. |
| Software | R (MatchIt, WeightIt, cobalt, and survey packages). |

**B. Covariate balance before and after matching/weighting (standardized mean differences)**

| Variable | Unadjusted | After PSM | After IPTW/ATE | After overlap weighting |
| --- | --- | --- | --- | --- |
| Center B (vs Center A) | 0.030 | 0.000 | −0.137 | 0.000 |
| Uyghur | 1.030 | 0.000 | −0.077 | 0.000 |
| Han | −0.887 | 0.000 | 0.047 | 0.000 |
| Kazakh | −0.188 | 0.000 | 0.031 | 0.000 |
| Other ethnicity | −0.271 | 0.000 | 0.038 | 0.000 |
| Female | −0.094 | −0.108 | −0.010 | 0.000 |
| Age, months | −0.129 | −0.052 | 0.208 | 0.000 |

*SMD, standardized mean difference; PSM, propensity score matching; IPTW, inverse probability of treatment weighting; ATE, average treatment effect. After matching, study center and ethnicity were exactly balanced by exact matching; the largest residual SMD was 0.108 for sex, marginally above the conventional 0.1 threshold, and was further controlled by covariate adjustment for sex in the outcome model. After IPTW, residual imbalance remained for age (SMD 0.208) and study center (SMD −0.137), which was likewise controlled by covariate adjustment in the weighted outcome model; the effective sample size after IPTW was 503 cases and 11,630 controls. Overlap weighting achieved near-exact balance for all covariates by construction.*

**C. Associations of HAZ and BAZ with pediatric urolithiasis in matched and weighted analyses**

| Analysis | Exposure | OR (95% CI) | P |
| --- | --- | --- | --- |
| Propensity score matched sample | HAZ | 0.668 (0.643, 0.694) | <0.001 |
| Propensity score matched sample | BAZ | 0.705 (0.674, 0.737) | <0.001 |
| IPTW/ATE weighted joint model | HAZ | 0.615 (0.578, 0.656) | <0.001 |
| IPTW/ATE weighted joint model | BAZ | 0.774 (0.726, 0.826) | <0.001 |
| Overlap/ATO weighted joint model | HAZ | 0.590 (0.567, 0.615) | <0.001 |
| Overlap/ATO weighted joint model | BAZ | 0.691 (0.666, 0.718) | <0.001 |

*ORs correspond to each 1-unit increase in HAZ or BAZ. All models included HAZ and BAZ simultaneously and were adjusted for study center, ethnicity, sex, and age (months). Matched-sample estimates used standard errors clustered on matched pairs; weighted estimates used robust (sandwich) standard errors. OR, odds ratio; CI, confidence interval; HAZ, height-for-age Z-score; BAZ, BMI-for-age Z-score; IPTW, inverse probability of treatment weighting; ATE, average treatment effect; ATO, average treatment effect in the overlap population.*

**Table S8. Restricted cubic spline knot locations and tests for overall and nonlinear associations.**

*A. Knot locations. Restricted cubic splines used four knots at the 5th, 35th, 65th, and 95th percentiles.*

| Variable | Knot number | Percentile | Knot location |
| --- | --- | --- | --- |
| HAZ | 1 | 5th | -2.360 |
| HAZ | 2 | 35th | -0.130 |
| HAZ | 3 | 65th | 0.930 |
| HAZ | 4 | 95th | 2.647 |
| BAZ | 1 | 5th | -2.180 |
| BAZ | 2 | 35th | -0.290 |
| BAZ | 3 | 65th | 0.860 |
| BAZ | 4 | 95th | 2.940 |

*B. Overall and nonlinear tests from RCS models.*

| Model | Variable | Test | df | Chi-square | *P* |
| --- | --- | --- | --- | --- | --- |
| Primary RCS | HAZ | Overall | 3 | 814.703 | <0.001 |
| Primary RCS | HAZ | Nonlinear | 2 | 142.749 | <0.001 |
| Primary RCS | BAZ | Overall | 3 | 211.235 | <0.001 |
| Primary RCS | BAZ | Nonlinear | 2 | 0.326 | 0.850 |
| Joint RCS adjusted for BAZ | HAZ | Overall | 3 | 925.252 | <0.001 |
| Joint RCS adjusted for BAZ | HAZ | Nonlinear | 2 | 98.614 | <0.001 |
| Joint RCS adjusted for HAZ | BAZ | Overall | 3 | 371.558 | <0.001 |
| Joint RCS adjusted for HAZ | BAZ | Nonlinear | 2 | 6.277 | 0.043 |

**Table S9. Subgroup-specific associations between HAZ and pediatric urolithiasis.**

*ORs correspond to each 1-unit increase in HAZ. Subgroup-specific models were adjusted for covariates not used for stratification.*

| Subgroup | Level | OR (95% CI) | *P* | n | Cases | Controls |
| --- | --- | --- | --- | --- | --- | --- |
| center | Center A | 0.677 (0.650, 0.705) | <0.001 | 9613 | 1468 | 8145 |
| center | Center B | 0.563 (0.528, 0.600) | <0.001 | 4414 | 712 | 3702 |
| gender | Male | 0.656 (0.628, 0.684) | <0.001 | 9086 | 1329 | 7757 |
| gender | Female | 0.610 (0.576, 0.646) | <0.001 | 4941 | 851 | 4090 |
| ethnicity | Uyghur | 0.630 (0.607, 0.653) | <0.001 | 8122 | 2019 | 6103 |
| ethnicity | Han | 0.674 (0.569, 0.803) | <0.001 | 4257 | 72 | 4185 |
| ethnicity | Kazakh | 0.765 (0.651, 0.900) | 0.001 | 912 | 66 | 846 |
| ethnicity | Other | 0.492 (0.364, 0.657) | <0.001 | 736 | 23 | 713 |
| age_group | <36 months | 0.765 (0.715, 0.817) | <0.001 | 1695 | 618 | 1077 |
| age_group | 36-70 months | 0.617 (0.573, 0.664) | <0.001 | 4179 | 427 | 3752 |
| age_group | 71-142 months | 0.637 (0.603, 0.673) | <0.001 | 6688 | 798 | 5890 |
| age_group | >=143 months | 0.572 (0.516, 0.633) | <0.001 | 1465 | 337 | 1128 |
| baz_cat3 | Normal | 0.558 (0.531, 0.586) | <0.001 | 8735 | 1486 | 7249 |
| baz_cat3 | Thinness | 0.821 (0.745, 0.902) | <0.001 | 856 | 258 | 598 |
| baz_cat3 | Overweight/obesity | 0.592 (0.556, 0.630) | <0.001 | 4436 | 436 | 4000 |

**Table S10. Subgroup-specific associations between BAZ and pediatric urolithiasis.**

*ORs correspond to each 1-unit increase in BAZ. Subgroup-specific models were adjusted for covariates not used for stratification.*

| Subgroup | Level | OR (95% CI) | *P* | n | Cases | Controls |
| --- | --- | --- | --- | --- | --- | --- |
| center | Center A | 0.792 (0.761, 0.823) | <0.001 | 9613 | 1468 | 8145 |
| center | Center B | 0.782 (0.739, 0.826) | <0.001 | 4414 | 712 | 3702 |
| gender | Male | 0.787 (0.757, 0.818) | <0.001 | 9086 | 1329 | 7757 |
| gender | Female | 0.795 (0.752, 0.840) | <0.001 | 4941 | 851 | 4090 |
| ethnicity | Uyghur | 0.785 (0.759, 0.812) | <0.001 | 8122 | 2019 | 6103 |
| ethnicity | Han | 0.845 (0.724, 0.985) | 0.032 | 4257 | 72 | 4185 |
| ethnicity | Kazakh | 0.780 (0.659, 0.923) | 0.004 | 912 | 66 | 846 |
| ethnicity | Other | 0.800 (0.606, 1.050) | 0.111 | 736 | 23 | 713 |
| age_group | <36 months | 0.781 (0.721, 0.844) | <0.001 | 1695 | 618 | 1077 |
| age_group | 36-70 months | 0.853 (0.797, 0.911) | <0.001 | 4179 | 427 | 3752 |
| age_group | 71-142 months | 0.750 (0.713, 0.788) | <0.001 | 6688 | 798 | 5890 |
| age_group | >=143 months | 0.740 (0.670, 0.816) | <0.001 | 1465 | 337 | 1128 |
| haz_cat3 | Non-stunting | 0.740 (0.714, 0.767) | <0.001 | 13097 | 1737 | 11360 |
| haz_cat3 | Moderate stunting | 0.697 (0.612, 0.788) | <0.001 | 553 | 255 | 298 |
| haz_cat3 | Severe stunting | 0.763 (0.664, 0.871) | <0.001 | 377 | 188 | 189 |

**Table S11. Subgroup interaction P values with multiple-testing correction.**

*FDR indicates false discovery rate correction using the Benjamini-Hochberg method.*

| Exposure | Subgroup | *P* interaction | FDR-adjusted *P* | Bonferroni-adjusted *P* |
| --- | --- | --- | --- | --- |
| HAZ | center | <0.001 | <0.001 | <0.001 |
| HAZ | gender | 0.108 | 0.181 | 1.000 |
| HAZ | ethnicity | 0.058 | 0.116 | 0.581 |
| HAZ | age_group | <0.001 | <0.001 | <0.001 |
| HAZ | baz_cat3 | <0.001 | <0.001 | <0.001 |
| BAZ | center | 0.179 | 0.255 | 1.000 |
| BAZ | gender | 0.753 | 0.753 | 1.000 |
| BAZ | ethnicity | 0.617 | 0.686 | 1.000 |
| BAZ | age_group | 0.020 | 0.049 | 0.197 |
| BAZ | haz_cat3 | 0.559 | 0.686 | 1.000 |

**Supplementary Figures**

**Figure S1. Joint restricted cubic spline sensitivity analyses of HAZ and BAZ.** Restricted cubic spline sensitivity analyses were performed with mutual adjustment of HAZ and BAZ. Four knots were placed at the 5th, 35th, 65th, and 95th percentiles. Odds ratios were estimated using a z-score of 0 as the reference value. Shaded areas represent 95% confidence intervals. (A) Association between HAZ and pediatric urolithiasis after additional adjustment for BAZ. (B) Association between BAZ and pediatric urolithiasis after additional adjustment for HAZ. Abbreviations: BAZ, BMI-for-age Z-score; CI, confidence interval; HAZ, height-for-age Z-score; OR, odds ratio; RCS, restricted cubic spline.

**Figure S2. Covariate balance before and after propensity score matching.** The love plot shows standardized mean differences of covariates before and after propensity score matching. The propensity score model included study center, ethnicity, sex, and age. HAZ and BAZ were not included in the propensity score model because they were the primary exposure variables. The vertical reference line indicates an absolute standardized mean difference of 0.1, which was used as the threshold for acceptable balance. Abbreviations: BAZ, BMI-for-age Z-score; HAZ, height-for-age Z-score; PSM, propensity score matching; SMD, standardized mean difference.

**Figure S3. Covariate balance before and after overlap weighting.** The love plot shows standardized mean differences of covariates before and after propensity score overlap weighting. The propensity score model included study center, ethnicity, sex, and age. HAZ and BAZ were not included in the propensity score model because they were the primary exposure variables. The vertical reference line indicates an absolute standardized mean difference of 0.1. Abbreviations: BAZ, BMI-for-age Z-score; HAZ, height-for-age Z-score; SMD, standardized mean difference.

**Figure S4. Covariate balance before and after inverse probability of treatment weighting.** The love plot shows standardized mean differences of covariates before and after inverse probability of treatment weighting. The propensity score model included study center, ethnicity, sex, and age. HAZ and BAZ were not included in the propensity score model because they were the primary exposure variables. The vertical reference line indicates an absolute standardized mean difference of 0.1. Abbreviations: ATE, average treatment effect; BAZ, BMI-for-age Z-score; HAZ, height-for-age Z-score; IPTW, inverse probability of treatment weighting; SMD, standardized mean difference.

**Table S12. Item-level missing data in the retained analytic sample before imputation (N = 14,027)**

| **Domain** | **Variable** | **Missing, n (%)** | **Center A, n** | **Center B, n** |
| --- | --- | --- | --- | --- |
| Core (outcome, exposures, demographics) | Case status (outcome) | 0 (0.00%) | 0 | 0 |
| Core (outcome, exposures, demographics) | Height-for-age Z-score (HAZ) | 273 (1.95%) | 227 | 46 |
| Core (outcome, exposures, demographics) | BMI-for-age Z-score (BAZ) | 361 (2.57%) | 275 | 86 |
| Core (outcome, exposures, demographics) | Sex | 0 (0.00%) | 0 | 0 |
| Core (outcome, exposures, demographics) | Ethnicity | 0 (0.00%) | 0 | 0 |
| Core (outcome, exposures, demographics) | Age (months) | 0 (0.00%) | 0 | 0 |
| Urinalysis | Urinary protein | 4621 (32.94%) | 1652 | 2969 |
| Urinalysis | Urinary occult blood | 75 (0.53%) | 75 | 0 |
| Urinalysis | Urobilinogen | 4473 (31.89%) | 1653 | 2820 |
| Urinalysis | Urinary bilirubin | 4473 (31.89%) | 1653 | 2820 |
| Urinalysis | Urinary glucose | 4472 (31.88%) | 1652 | 2820 |
| Urinalysis | Urinary ketone bodies | 4473 (31.89%) | 1653 | 2820 |
| Urinalysis | Urine sediment | 26 (0.19%) | 26 | 0 |
| Urinalysis | Urine specific gravity | 4427 (31.56%) | 1662 | 2765 |
| Urinalysis | Urine pH | 4427 (31.56%) | 1661 | 2766 |
| Hematology | Red blood cell count | 106 (0.76%) | 99 | 7 |
| Hematology | Mean corpuscular volume | 26 (0.19%) | 26 | 0 |
| Hematology | Mean corpuscular hemoglobin concentration | 69 (0.49%) | 54 | 15 |
| Hematology | Mean corpuscular hemoglobin | 32 (0.23%) | 26 | 6 |
| Hematology | Red cell distribution width-CV | 26 (0.19%) | 26 | 0 |
| Hematology | Hematocrit | 59 (0.42%) | 52 | 7 |
| Hematology | Hemoglobin | 40 (0.29%) | 26 | 14 |
| Hematology | Platelet count | 61 (0.43%) | 59 | 2 |
| Hematology | Mean platelet volume | 55 (0.39%) | 50 | 5 |
| Hematology | Platelet distribution width | 55 (0.39%) | 50 | 5 |
| Hematology | Platelet-large cell ratio | 55 (0.39%) | 50 | 5 |
| Biochemistry | Alanine aminotransferase | 93 (0.66%) | 85 | 8 |
| Biochemistry | Aspartate aminotransferase | 92 (0.66%) | 84 | 8 |
| Biochemistry | Gamma-glutamyl transferase | 582 (4.15%) | 206 | 376 |
| Biochemistry | Direct bilirubin | 1837 (13.10%) | 321 | 1516 |
| Biochemistry | Total bilirubin | 95 (0.68%) | 87 | 8 |
| Biochemistry | Albumin | 94 (0.67%) | 83 | 11 |
| Biochemistry | Indirect bilirubin | 112 (0.80%) | 103 | 9 |
| Biochemistry | Total protein | 106 (0.76%) | 83 | 23 |
| Biochemistry | Globulin | 94 (0.67%) | 83 | 11 |
| Biochemistry | Creatinine | 89 (0.63%) | 77 | 12 |
| Biochemistry | Blood urea nitrogen | 87 (0.62%) | 77 | 10 |
| Biochemistry | Uric acid | 90 (0.64%) | 77 | 13 |
| Electrolytes/minerals | Potassium | 90 (0.64%) | 77 | 13 |
| Electrolytes/minerals | Chloride | 85 (0.61%) | 77 | 8 |
| Electrolytes/minerals | Sodium | 86 (0.61%) | 78 | 8 |
| Electrolytes/minerals | Magnesium | 3814 (27.19%) | 2953 | 861 |
| Electrolytes/minerals | Calcium | 2939 (20.95%) | 2806 | 133 |
| Electrolytes/minerals | Cholinesterase | 3743 (26.68%) | 2215 | 1528 |
| Coagulation | Prothrombin time-INR | 349 (2.49%) | 321 | 28 |
| Coagulation | Prothrombin time | 349 (2.49%) | 321 | 28 |
| Coagulation | Prothrombin time ratio | 824 (5.87%) | 771 | 53 |
| Coagulation | Prothrombin activity | 349 (2.49%) | 321 | 28 |
| Coagulation | Thrombin time | 1295 (9.23%) | 1216 | 79 |
| Coagulation | Activated partial thromboplastin time | 349 (2.49%) | 321 | 28 |
| Coagulation | Fibrinogen | 349 (2.49%) | 321 | 28 |
| Coagulation | D-dimer | 2001 (14.27%) | 992 | 1009 |

**Table S13. Diagnostic performance of the multivariable logistic regression models (outcome: pediatric urolithiasis; N = 14,027; 2,180 events)**

| **Metric** | **Model 1 (HAZ + BAZ)** | **Model 2 (+ sex, ethnicity, age in months)** | **Model 3 (+ study center; primary model)** |
| --- | --- | --- | --- |
| C-statistic (AUC) | 0.773 | 0.833 | 0.834 |
| AUC 95% CI (DeLong) | 0.762–0.784 | 0.825–0.842 | 0.826–0.843 |
| Calibration intercept* | 0.000 | 0.000 | 0.000 |
| Calibration slope* | 1.000 | 1.000 | 1.000 |
| Brier score | 0.112 | 0.103 | 0.102 |
| Hosmer–Lemeshow χ² (df = 8) | 24.64 | 17.75 | 32.57 |
| Hosmer–Lemeshow P | 0.002 | 0.023 | <0.001 |
| AIC | 10,291.6 | 9,265.5 | 9,250.3 |
| BIC | 10,314.2 | 9,325.9 | 9,318.2 |
| Parameters (excl. intercept) | 2 | 7 | 8 |
| Events per variable (EPV) | 1,090.0 | 311.4 | 272.5 |

**Calibration intercept and slope were obtained by regressing the observed outcome on the model's linear predictor. These are apparent (in-sample) values; an intercept of 0 and a slope of 1 are expected by construction for maximum-likelihood logistic models evaluated on their training data, and optimism-corrected values would require internal validation (e.g., bootstrap). The Hosmer–Lemeshow test is highly sensitive to trivial deviations in very large samples and is reported for reference only. EPV = 2,180 events divided by the number of estimated parameters. AUC, area under the receiver operating characteristic curve; AIC, Akaike information criterion; BIC, Bayesian information criterion.*
